# Swim bladder-derived biomaterials: structures, compositions, properties, modifications, and biomedical applications

**DOI:** 10.1186/s12951-024-02449-w

**Published:** 2024-04-17

**Authors:** Xiaorong Lan, Mingdong Luo, Meiling Li, Linpeng Mu, Guangwen Li, Gong Chen, Zhoukun He, Jingang Xiao

**Affiliations:** 1 Luzhou Key Laboratory of Oral & Maxillofacial Reconstruction and Regeneration, The Affiliated Stomatological Hospital, Southwest Medical University, Luzhou, 646000 China; 2 Metabolic Vascular Diseases Key Laboratory of Sichuan Province, Southwest Medical University, Luzhou, 646000 China; 3 Basic Medicine Research Innovation Center for Cardiometabolic Diseases, Ministry of Education, Southwest Medical University, Luzhou, 646000 China; 4 Institute of Stomatology, Southwest Medical University, Luzhou, 646000 China; 5 Southwest Hospital of Army Military Medical University, Chongqing, 400038 China; 6 Institute for Advanced Study, Research Center of Composites & Surface and Interface Engineering, Chengdu University, Chengdu, 610106 China; 7 Department of Cardiology, The Affiliated Hospital, Southwest Medical University, Luzhou, 646000 China

**Keywords:** Swim bladder, Tissue repair, Cardiovascular repair, Hydrogel, Biological adhesive

## Abstract

Animal-derived biomaterials have been extensively employed in clinical practice owing to their compositional and structural similarities with those of human tissues and organs, exhibiting good mechanical properties and biocompatibility, and extensive sources. However, there is an associated risk of infection with pathogenic microorganisms after the implantation of tissues from pigs, cattle, and other mammals in humans. Therefore, researchers have begun to explore the development of non-mammalian regenerative biomaterials. Among these is the swim bladder, a fish-derived biomaterial that is rapidly used in various fields of biomedicine because of its high collagen, elastin, and polysaccharide content. However, relevant reviews on the biomedical applications of swim bladders as effective biomaterials are lacking. Therefore, based on our previous research and in-depth understanding of this field, this review describes the structures and compositions, properties, and modifications of the swim bladder, with their direct (including soft tissue repair, dural repair, cardiovascular repair, and edible and pharmaceutical fish maw) and indirect applications (including extracted collagen peptides with smaller molecular weights, and collagen or gelatin with higher molecular weights used for hydrogels, and biological adhesives or glues) in the field of biomedicine in recent years. This review provides insights into the use of swim bladders as source of biomaterial; hence, it can aid biomedicine scholars by providing directions for advancements in this field.

## Introduction

Regenerative biomaterials, an important subfield of biomedical materials, belong to class III medical device products [1, 2]. These products are mainly used to treat, repair, and replace human tissues and organs, or to enhance their functions. It is one of the most extensive interdisciplinary fields in contemporary science and technology, involving materials science, biology, and medicine, and is an important foundation of biotechnology and biomedical engineering, the two pillars of modern medicine. Based on their sources, tissue-derived regenerative biomaterials can be classified into autologous, allogeneic, and xenogeneic [3–5]. Autologous tissue transplantation has good histocompatibility with no rejection because it is taken from the patient; additionally, it does not increase the economic burden of the patient [6]. However, because the tissue source is limited, this personalized therapy cannot be employed in extensive treatments and can cause trauma at the source site, affecting the patient's physiological condition. This method is similar to "robbing Peter to pay Paul," which does not achieve a comprehensive solution to the problem and is limited in therapeutic use. Allogeneic tissues are usually sourced from cadavers; however, their use is hindered by immune reactions causing rejection and premature absorption in situ [7–10]. In addition, the lack of laws and regulations and public awareness of organ donation limit the availability of the material. Xenogeneic tissue, also known as animal-derived material, mainly includes animal skin, bone collagen, and membranes, and is processed into various products that can replace certain organs or functions of the human body [11]. Xenogeneic tissues are mainly obtained from animals such as pigs and cattle. Through decellularization treatment, their rejection post-transplantation is reduced; hence, a wide range of sources can be explored [11–13]. Furthermore, xenogeneic tissues show good histocompatibility owing to their compositional and structural similarities with human tissues and organs [14]. Additionally, this type of material exhibits good mechanical and degradation compliance [15]. Implants can act as a mechanical support at the affected site and replace defective tissues or organs by performing related functions. Later, they gradually degrade while the regeneration of new tissues or organs is induced and tissue regeneration and wound repair are ultimately achieved [16, 17]. These advantages allow the use of animal-derived biomaterials in numerous clinical applications and have gradually resulted in the expanded use of these materials in the field of regenerative medicine.

**Fig. 1:**
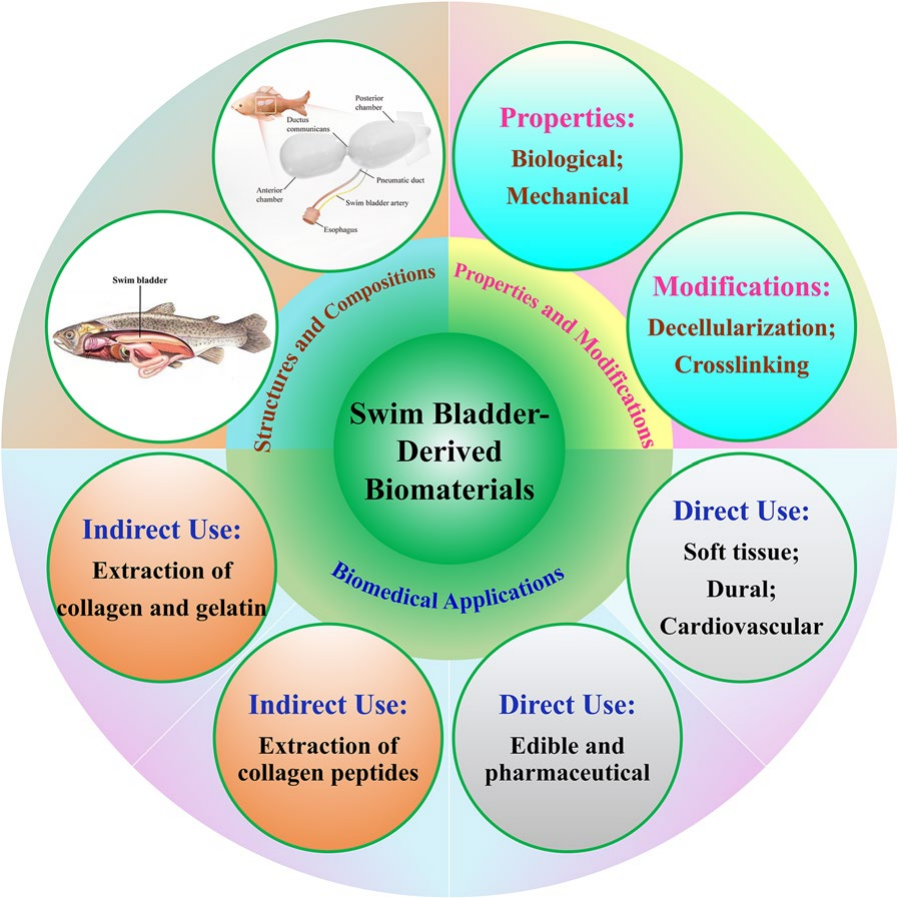
An overview of the structures, compositions, properties, modifications, and biomedical applications of swim bladder-derived biomaterials

To date, a variety of animal-derived biomedical materials and products have been successfully developed, such as absorbable biofilms for oral use [18], artificial dural patches [19, 20] based on porcine small intestinal submucosa, and various bioprosthetic heart valve products [20, 21] based on porcine or bovine pericardium. However, there may be a risk of infection with pathogenic microorganisms after implantation of tissues from pigs, cattle, and other mammals [22, 23]. Therefore, researchers have begun to explore the development of non-mammalian biological renewable materials as alternatives. Among these, fish-derived biomaterials, without the limitation of national and religious beliefs, such as the swim bladder and fish collagen, have gradually gained attention [24–30]. Meanwhile, the swim bladder, as an aquatic by-product, is relatively inexpensive than the tissue from pigs, cattle, and other mammals, and it does not carry the risk of land-based infectious diseases found in cattle such as foot-and-mouth disease (FMD), bovine spongiform encephalopathy (BSE), mad cow disease, or other prion diseases [31]. Therefore, swim bladder-derived constituents have been widely considered in biomedicine, cosmetics, food, and other fields as new biomaterials [24–27], such as fish collagen [28–30], fish gelatin [32–35], biological adhesives or glues [36, 37], hair cosmetics [38], fabrics [39], bio-piezoelectric separators [40], nanogenerators [41, 42], mini-generators [43], environmental actors [44], sensor biofilm matrices [45], and various extracellular matrix (ECM) products [46–48]. This review summarizes recent publications on swim bladder-derived biomaterials, as shown in Fig. 1, based on their structures, compositions, properties, modifications, and the latest direct or indirect applications in the biomedical field.

**Fig. 2:**
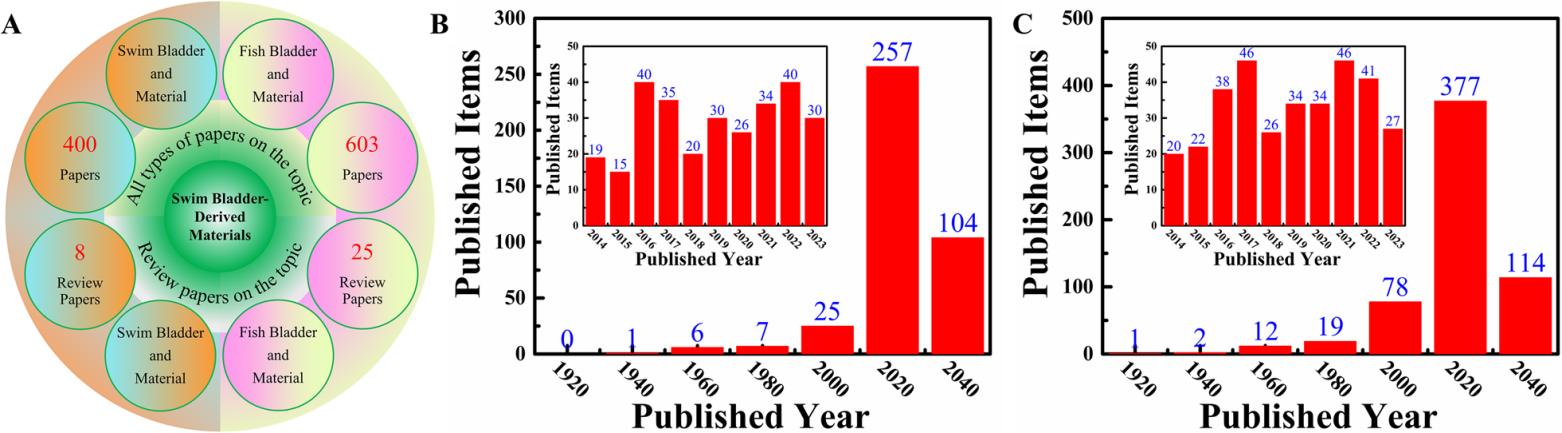
Summary of relevant reports on swim bladder-derived materials retrieved from Web of Science (WOS) on the topic, among which the number of review papers is counted (**A**, January 1, 1900–December 31, 2023). Statistical data of publications per 20-year span containing the words "swim bladder" **B** or "fish bladder" **C** and "material" (January 1, 1900–December 31, 2023), and the data between January 1, 2014, and December 31, 2023 (inserted images in B and C)

According to search results from the Web of Science (WOS, January 1, 1900–December 31, 2023), there are only 1,003 reports on swim bladder-derived biomaterials. As shown in Fig. 2A, only 400 papers, of which 8 are review papers, can be obtained with "swim bladder" and "material" as search terms on the topic, whereas only 603 papers, of which 25 are review papers, can be obtained with "fish bladder" and "material" as search terms on the topic. As shown in Fig. 2B, C, the publication data show that this research topic has been gaining attention, especially over the last 10 years (a summary of 289 out of 400 reports, and 334 out of 603 reports). Among the review publications, only five reviews related to "swim bladder" or "fish bladder" [35, 49–52]. However, the structures, compositions, properties, modifications, and biomedical applications of swim bladder-derived biomaterials have not been systematically reviewed. This paper summarizes the literatures on swim bladder-derived biomaterials, which will aid researchers in understanding the importance of swim bladder-derived biomaterials and make important contributions for advancing this field.

## Structures and compositions of the swim bladder

**Fig. 3:**
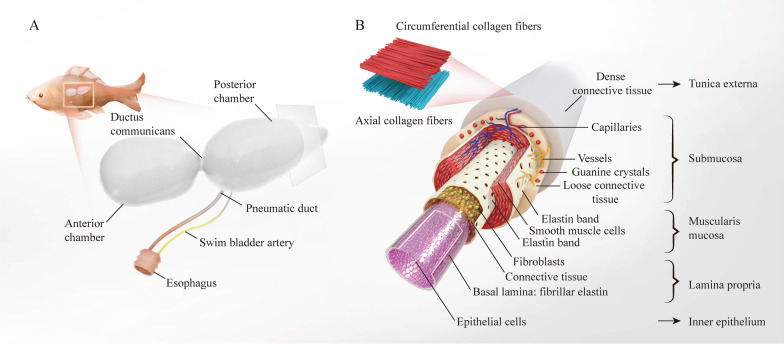
Shape, composition, and relative position of the swim bladder in fish (**A**). Reproduced with permission from Ref. [53]. Copyright 2006, Wiley-Liss, Inc. Scheme of the structures and compositions of the posterior chambers (**B**)

The swim bladder, commonly known as the fish maw, is an important buoyancy organ in bony fish. It is a long thin sac located at the back of the body cavity, accounting for approximately 5% of the body volume of the fish (Fig. 3A) [53], consisting of two chambers, the front and back, with a sphincter between them that regulates the flow of air between the chambers [54]. The front chamber is connected to the inner ear by a string of small bones called the Weberian ossicle, which is specialized for the perception of sound, whereas the back chamber functions mainly as a hydrostatic organ [55]. The chamber contains oxygen, nitrogen, and carbon dioxide. Contraction and expansion of the swim bladder can be regulated by the swim bladder muscle to adjust the density of the body to rise or sink in the water column [56].

The anterior and posterior chambers of the swim bladders of different fish species are similar in composition and structure. For example, in the toadfish (*Opsanus tau L.*), the wall of the anterior and posterior chambers of the swim bladder consists of several thin layers of tissue; from the inside to the outside, these are: inner epithelium, lamina propria, muscularis mucosa, submucosa, and tunica externa (Fig. 3B) [57, 58]. The inner epithelium is a monolayer of flattened epithelial cells covered with short microvilli and joined by desmosomes. The lamina propria is distributed between the inner epithelium and muscularis mucosa and consists of the basal lamina, connective tissue, and fibroblasts. The basal lamina is close to the inner epithelium and is composed of a thin layer of highly fibrillar elastin, whereas the connective tissue is close to the muscularis mucosa and is composed of a wider layer of thick collagen fibers and fibroblasts. The muscularis mucosa consists of a wide layer of smooth muscle cells between two continuous elastin bands that separate the muscularis mucosa from the lamina propria and submucosa. In addition, the muscularis mucosa contains collagen fibers, small elastic fibers, a few fibroblasts, and nerves. The submucosa consists of loose connective tissue with more capillaries, vessels, and guanine crystals, some of which may also contain lipid membranes, making the swim bladder wall airtight and resistant to gas leakage. The tunica externa is a dense layer of connective tissue mainly composed of thick collagen fibrils, which vary in diameter. In this layer, the dense collagen fibril is arranged in two layers, with the parallel fibers of each layer arranged perpendicular to those of the other layer [59].

The swim bladder is composed of high-value substances such as collagen, elastin, and polysaccharides [47, 60]. Collagen and elastin make up collagen and elastic fibers, respectively. Collagen in the swim bladder is type I collagen [61], which consists of two α1 and one α2 chains, all of which are left-handed polypeptide chains, arranged in a right-handed triple helix structure. Each chain contains approximately 1,014 amino acids, among which glycine is the most abundant (30%), followed by proline, hydroxyproline, threonine, methionine, isoleucine, and phenylalanine [60, 62]. The composition of swim bladder collagen (SBC) is similar to that of traditional bovine pericardium collagen [12], but the amino acid content in different fish species differs from that in mammals. For example, the amounts of threonine, methionine, isoleucine, and phenylalanine in *Silver carp* SBC are higher than those in bovine pericardium, but the amounts of other amino acids are similar or lower than those in bovine pericardium [60, 63].

SBC maintains its structural stability through covalent crosslinking of the glycerin triplet with hydrogen bonds. Meanwhile, proline has a metastable conformation, endowing collagen with good flexibility [64]. The thermal transformation of swim bladder-derived collagen occurs at approximately 35 °C. Because of the low imine content extracted by aquatic organisms, thermal denaturation generally occurs at a slightly lower temperature than that required for the denaturation of mammalian collagen, but higher than that required for the denaturation of collagen extracted from fish skin, fish scales, and other internal organs [65–67]. There are different methods for extracting collagen from swim bladder, including pepsin dissolution, chemical (acid, alkali, and salt) dissolution, and fermentation [68, 69]. Collagen yield varies from 30 to 60% based on the species and methods of isolation [31, 70, 71]. Owing to the presence of specific peptides and amino acids, fish collagen hydrolysates have pharmacological benefits, such as anti-inflammatory, antibacterial, antitumor, antiaging, and free radical scavenging activities [72, 73].

A large amount of elastin is found in the muscularis mucosa of the swim bladder wall with an amino acid composition similar to that of elastin from mammals. The main difference is that fish elastin contains a higher proportion of polar amino acids, although the contents of alanine and valine are slightly lower [74]. Polysaccharides are also important functional components of the swim bladder, and their content is up to 10 wt% [75]. They have been shown to promote wound healing and prevent infection and thrombosis [76, 77]. The main polysaccharides in the swim bladder are chondroitin sulfate (95%, type A/C and type B at a ratio of 1.4:1) and hyaluronic acid (HA; 5%) [78]; chondroitin sulfate plays an important role in wound healing [79, 80]. In recent years, chondroitin sulfate has been used for cartilage repair and tissue engineering [81]. In addition, fish swim bladders are rich in inorganic salts and vitamins, particularly Ca, followed by K and Mg, as well as in trace elements such as Zn, Cu, Fe, and Se. These trace elements are essential for maintaining normal physiological functions and health in the human body [82].

## Properties of the swim bladder

### Biological properties

For swim bladders to be considered effective biomaterials with no adverse properties, they should meet the corresponding national standards for biomaterials. Hence, testing their biological characteristics is essential. Cytotoxicity and hemocompatibility are important components of biocompatibility testing. Cytotoxicity testing is often the first step in verifying the compatibility of biomaterials for use in humans [83]. Platelet activation and hemolysis tests verify the hemocompatibility of biomaterials [84], which is a prerequisite for direct blood contact applications of swim bladder-derived biomaterials.

The safety assessment of swim bladder has been performed according to the ISO 10993 test principles [84]. Fresh swim bladder was soaked in minimum essential medium containing 10% fetal bovine serum, and the extract containing material components were used for cell culture. Cell viability and morphology testing of the material suggested that it was not cytotoxic. In the platelet activation experiment, whole blood from healthy individuals was extracted before the assay. A low degree of platelet activation indicated that the swim bladder-derived biomaterials did not cause coagulation. In the hemolysis test, physiological saline containing swim bladder extract was added to human blood, and if hemoglobin was detected without red blood cells, it indicated that the material did not cause hemolysis. Similarly, studies have shown that fresh swim bladder-derived biomaterial crosslinked using glutaraldehyde (GA) have no cytotoxicity and excellent hemocompatibility, thus confirming their biocompatibility [85, 86]. Additionally, swim bladder-derived biomaterials have demonstrated good biocompatibility in vivo. In a subcutaneous implantation experiment in rats, the degree of calcification of fish bladder material was lower than that of bovine pericardium material when both of them were crosslinked with GA [12]. In addition, the immune response induced by the fish bladder was weaker than that induced by the bovine pericardium. In rabbit dural repair experiments using carp swim bladder-derived biomaterial, fish swim bladder grafts healed well with the original dura, did not cause chronic or acute inflammatory reactions, and the degree of calcification was light [87]. Taken together, these observations suggest that swim bladder-derived biomaterials present good histocompatibility, anti-calcification ability, low immune response, and stable biological inertia.

### Mechanical properties

As a membranous structure, the swim bladder is mainly composed of dense and regular collagen fibers and dense elastic fibers distributed in collagen fibers [88]. However, the directionality of the arrangement of the internal fibers has a significant influence on its mechanical properties [89]. For example, the mechanical properties of the carp swim bladder [85], whether fresh or crosslinked with GA, were different in the circumferential and axial directions. The maximum tensile length, breaking strength, and elastic modulus of the swim bladder in the circumferential direction were larger than those in the axial direction [85]. The effects of the crosslinking treatment on the circumferential and axial mechanical properties were also different [85]. For example, the circumferential maximum tensile strength before and after crosslinking did not differ, but the axial maximum tensile strength increased after crosslinking. However, for the fracture strength and elastic modulus, the shear strength increased in the circumferential direction, whereas that in the axial direction showed little variation. This is because the mechanical strength was maximum when the fracture direction of the swim bladder was consistent with the arrangement direction of collagen fibers [85]. The collagen fibers in the swim bladder are mainly arranged in the circumferential direction, and collagen fibers are remarkably affected by crosslinking [85]. Moreover, differences in proline content of swim bladders from different fishes affect the content of collagen, causing changes in mechanical strength [86, 90]. Furthermore, the elastic modulus and mechanical properties of the swim bladder are very similar to those of human tissues such as the dura mater and aortic valve, suggesting its potential use as a substitute material for such tissues in clinical practice [87, 91].

## Modifications

### Decellularization

Decellularization refers to the removal of cells and other antigen molecules that may cause rejection in tissues or organs, while retaining the three-dimensional structure of the ECM (usually composed of collagen and elastin), glycosaminoglycans (GAGs), and functional proteins (proteoglycans and growth factors) [92, 93]. Thus, allogeneic or xenogeneic tissues or organs can be reused for repair and reconstruction after tissue injury in vivo and/or in vitro. To improve the efficiency of decellularization, physical, chemical, and biological methods are often combined [11, 94–107]. Generally, the decellularization step involves destroying the cell membrane using physical or chemical solvents, separating the cell components from the ECM using enzymes, and finally separating the cell fragments from the ECM [11].

Physical methods, such as stirring and oscillation, can be added to these steps to improve efficiency [100]. These methods mainly include ultrasound, ultra-high pressure, repeated freeze–thaw, and mechanical stirring [11, 98–102], and can destroy the cell membrane and release cell contents, which is conducive to the subsequent removal of cell debris using detergents. Physical methods cannot completely remove the cells and further treatment with chemical or biological agents is required [103].

Chemical methods mainly use one or several chemical reagents to alter the permeability of the cell membrane, which causes the cells to swell and rupture to achieve decellularization [11, 104]. Common chemical reagents include sodium deoxycholate, Triton X-100 and sodium dodecyl sulfate (SDS) [94, 103]. To improve the permeation efficiency of chemical reagents, they are typically combined with physical methods. However, depending on their structural characteristics and concentration, these reagents interact with proteins in the ECM resulting in varying degrees of damage or loss of ECM components [11].

Biological methods of decellularization mainly include the use of enzymes such as trypsin, dispase, and nuclease [105–107]. Enzymatic hydrolysis is the most effective in destroying the cell structure, but when the concentration of enzyme is too high or the reaction duration is too long, bioactive substances, including collagen and GAGs in the ECM, may be damaged. Therefore, it is necessary to determine the concentration, temperature, and duration of enzyme treatment based on the characteristics of the tissue involved.

Swim bladder, as a biomaterial, usually requires decellularization to considerably reduce its immunogenicity. Theoretically, the methods and principles of decellularization of biological tissues are similar, and can be used for swim bladders. For example, Bhanu et al. successfully prepared decellularized swim bladder grafts by decellularizing a fresh swim bladder with 1 M sodium chloride solution and 0.5% Triton X-100 under mild agitation [108]. Compared to fresh swim bladders, decellularized swim bladders have less tissue reactivity and antigenicity. In another study, to maximally preserve the three-dimensional fiber structure, Jalali et al. used a combination of physical (liquid nitrogen) and chemical (0.5% SDS) methods to decellularize the *whitefish* swim bladder, which was then cleaned with ethanol [46]. This produced a decellularized material with a well-preserved collagen fiber structure. Hematoxylin and eosin (H&E) staining showed that compared to cells observed in fresh swim bladder tissue, no cells were detected on the decellularized swim bladder-derived biomaterial, and the collagen fiber structure was well-preserved.

### Crosslinking

In biomedical applications, durability requirements differ depending on the body part. Temporary materials for soft tissue repair required for short-term use need not be durable. However, in permanent replacement implants, such as the dura mater and bioprosthetic heart valves, long-lasting materials are needed [47]. Thus, to meet these requirements, tissues usually need to be crosslinked to maximize their durability and extend their life. For swim bladder-derived biomaterials, GA is the main crosslinking agent currently used [85, 109–112], followed by 1, 4-butanediol diglycidyl ether (BDDGE) [113–115]. Moreover, swim bladder-derived biomaterials can be self-crosslinked, which is catalyzed by 1-(3-dimethylaminopropyl) -3-ethylcarbodiimide hydrochloride (EDC) and N-hydroxysuccinimide (NHS) [48, 116–121]. The mechanism, advantages, and disadvantages of the typical crosslinking methods are summarized in Table 1.

**Table 1:** Mechanism, advantages, and disadvantages of typical crosslinking methods

Crosslinking agents	Mechanism	Advantages	Disadvantages	Refs.
*GA*	Nucleophilic addition reaction between aldehyde groups in GA and amino groups in amino acid residues	Easy to use, low cost, low biodegradation, good biocompatibility and antithrombogenic, while maintaining integrity, strength, and elasticity; highest crosslinking degree and crosslinking stability among the three methods	The residual aldehyde groups are cytotoxic, which is not conducive to cell adhesion, growth, and endothelialization; they are also negatively-charged, which can lead to the adsorption of calcium ions, formation of calcium nuclei, and ultimately calcification	[85, 109–112]
*BDDGE*	The epoxy groups in BDDGE react with amino or carboxyl groups on collagen	The reaction is simple, mild, and efficient. The crosslinked tissue is lighter, whiter, and softer than GA. The inflammatory response is milder than GA	The crosslinking effect is not as good as that of GA. Resistance to degradation is slightly lower than GA, but is better than self-crosslinking	[113–115]
*Self-crosslinking*	Usually catalyzed by EDC and NHS, which can activate carboxyl groups on collagen, causing them to react with amino groups to form amide bonds	It requires mild reaction conditions, short reaction time, stable reaction products, and resists calcification. There are no residual crosslinking agents and no cytotoxicity issues	Compared to those of GA and BDDGE, self-crosslinking has the weakest crosslinking effect	[48, 116–121]

#### GA crosslinking

**Fig. 4:**
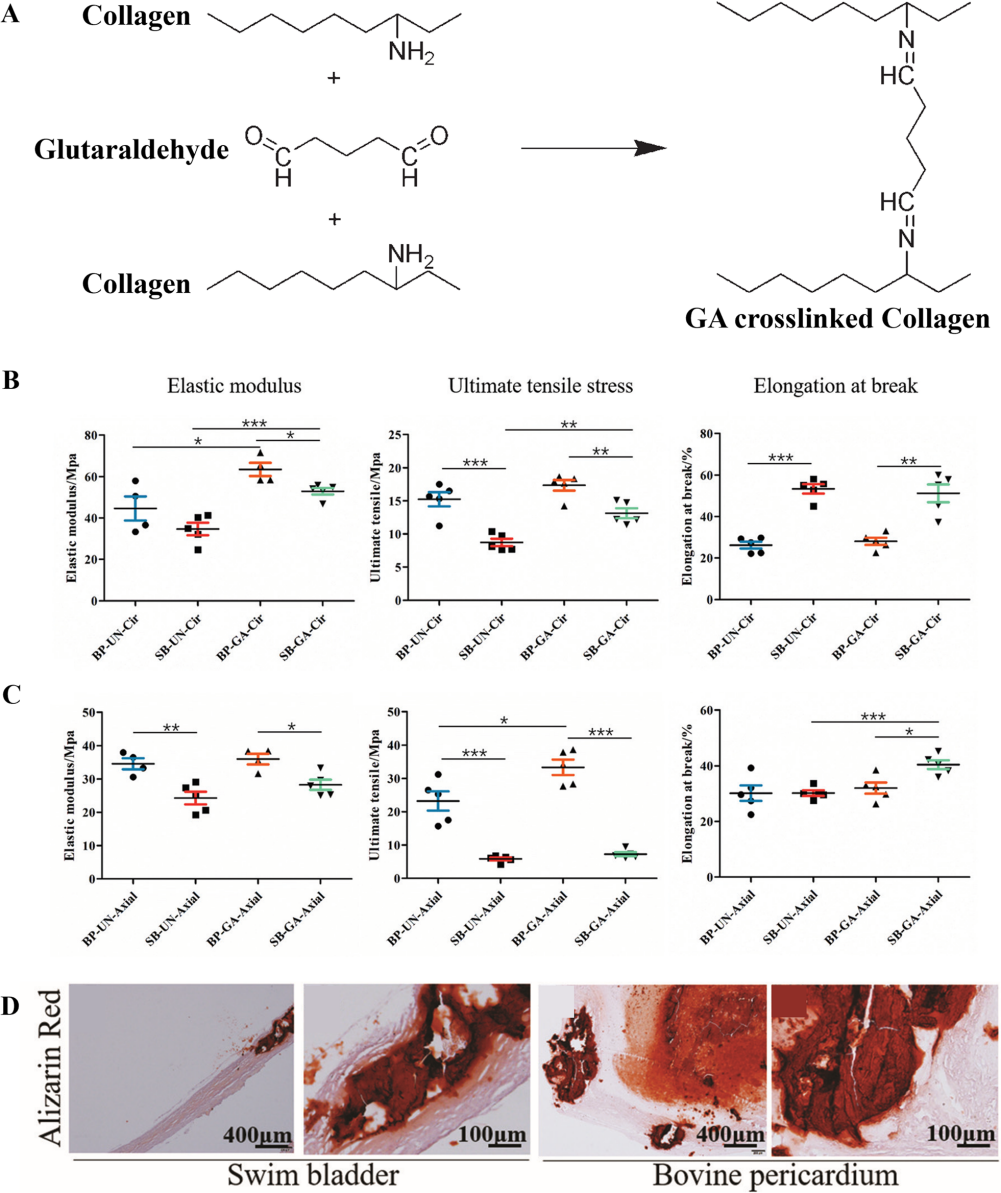
Schematic of the mechanism of GA crosslinking (**A**). Reprinted with permission from Ref. [111]. Copyright 2007, Wiley Periodicals, Inc. Mechanical properties of the swim bladder and bovine pericardium before and after crosslinking (**B**, **C**); Staining of tissue to show calcification after implantation into sheep pulmonary valve (**D**). B is the circumferential direction and C is the axial direction. BP, bovine pericardium; SB, swim bladder; UN, un-crosslinked; and GA, glutaraldehyde. The upper and lower borders of the box represent upper and lower quartiles, respectively. The horizontal line indicates the median value. Statistical significance is indicated as follows: *, p < 0.05; **, p < 0.01; and ***, p < 0.001. Reprinted with permission from Ref. [85]. Copyright 2021, Royal Society of Chemistry

GA is a bifunctional coupling agent with two aldehyde carbonyl groups and is soluble in water, where it exists as a monomer [109]. GA can connect peptide chains and proteins, and its crosslinking fixation of tissues can significantly reduce biodegradation and improve durability. In addition, the crosslinked material has good biocompatibility and antithrombogenic properties, while maintaining the integrity, strength, and elasticity of the anatomical structure [110]. However, the biocompatibility and thermal stability of other aldehydes used for crosslinking are weaker than those of GA; hence, GA crosslinking treatment is mainly used in clinical practice. The reaction mechanism involves the nucleophilic addition reaction between the aldehyde group of GA and the amino group of lysine or hydroxylysine (existing in collagen, elastin, glycoproteins, and proteoglycans), and then dehydration to a Schiff base that connects protein molecules, or aldehyde alcohol condensation between adjacent aldehydes, resulting in covalent crosslinking and fixation of biological tissues (Fig. 4A) [111].

Li et al. compared the mechanical properties of swim bladder and bovine pericardium after crosslinking with GA (Fig. 4B, C) [85]. They found that the elastic modulus and tensile strength were slightly lower, whereas the elongation at break was higher for the swim bladder in the circumferential and axial directions than that of the bovine pericardium, indicating that the swim bladder had stronger elasticity. Furthermore, calcification analysis after implantation into sheep pulmonary valve showed that the degree of calcification of the swim bladder was significantly lower than that of bovine pericardium tissue (Fig. 4D), thereby indicating that the GA-crosslinked swim bladder had better resistance to calcification.

GA crosslinking is advantageous for improving the mechanical strength of tissues and reducing their immunogenicity. However, the disadvantage is that the GA crosslinking method causes an incomplete reaction or dissociation of the residual aldehyde group, which can be released in vivo, causing cytotoxicity, and is not conducive to the adhesion of endothelial cells to form new active surfaces [112]. Therefore, non-GA crosslinking methods have been developed.

#### BDDGE crosslinking

**Fig. 5:**
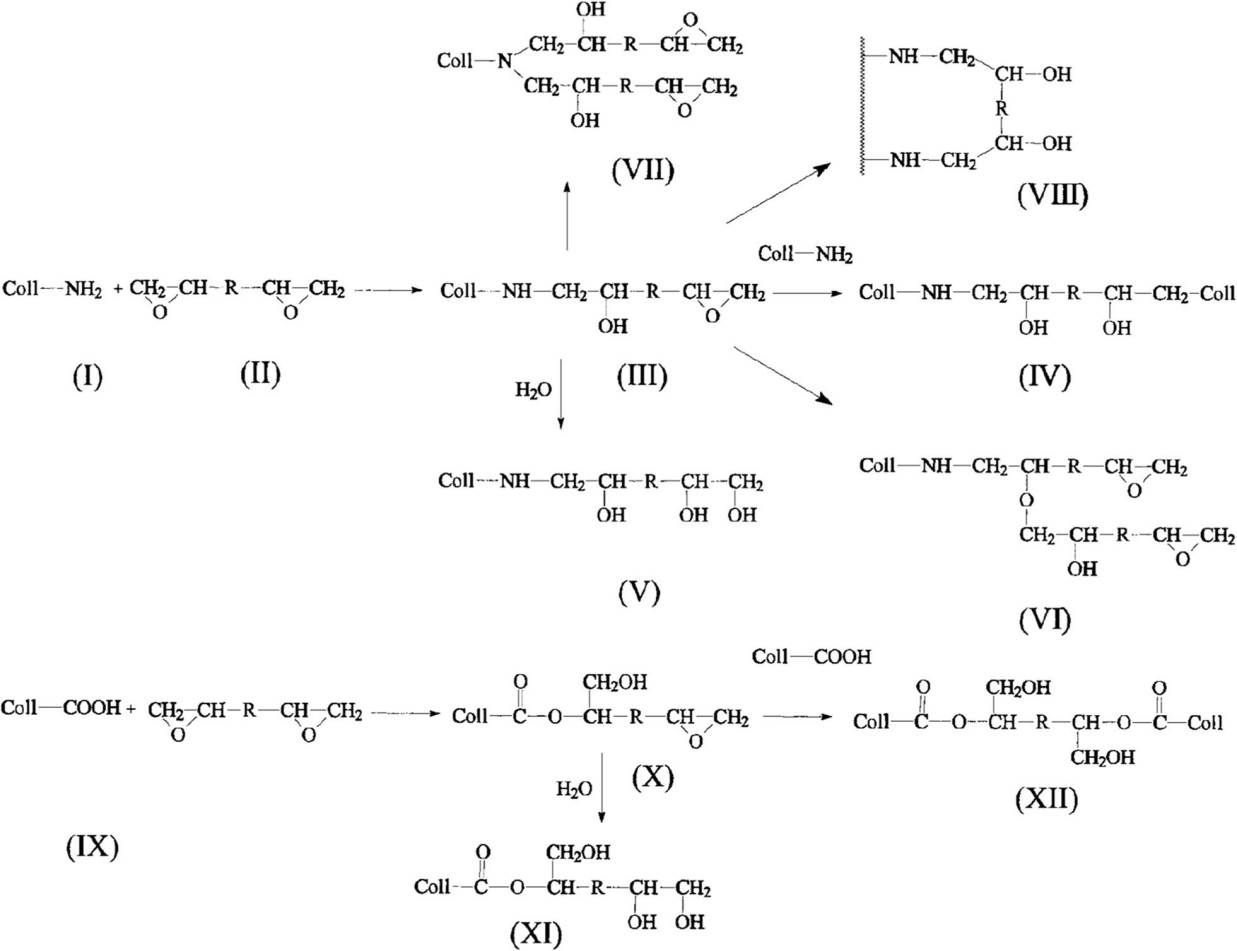
Schematic diagram of the reaction mechanism of BDDGE crosslinking. Reprinted with permission from Ref. [123]. Copyright 1999, John Wiley & Sons, Inc

BDDGE is formed by connecting two glycidyl ether groups with 1,4-butanediol. The epoxy groups at both ends are relatively active and can react with electrophilic and nucleophilic reagents. BDDGE is water-soluble and can be used as a bifunctional crosslinker to bind amino acids. It is widely used for the crosslinking of HA and gelatin [122]. Zeeman et al. studied its mechanism by crosslinking sheep skin with BDDGE (Fig. 5) [123]. At pH > 8.0, the reaction between the amino group of the (hydroxy)-lysine residue (I) and the epoxy group of diglycidyl ether (II) led to crosslinking between two adjacent helices through an intermediate (III) to form product (IV). At pH 9.0, after 160 h of crosslinking, the hydrolysis rate was below 6%; hence, hydrolysis under alkaline conditions had little effect on the reaction of epoxy, that is, reaction (III→V) had only a slight effect on the crosslinking process. Although the hydroxyl group formed in structure (III) could undergo an etherification reaction with another BDDGE molecule, resulting in structure (VI), the reaction only occurred under high temperature or tertiary amine catalysis; therefore, etherification and epoxy polymerization were not considered in practice. Additionally, the secondary amine in structure (III) could react with another BDDGE molecule to form (VII). Although the secondary amine after the first reaction could react with epoxy, its reaction rate was much lower than that of the primary amine, suggesting that the more likely reaction was for BDDGE to connect primary amines on the same or different peptide chains. If two amino groups of the collagen chain reacted with the same BDDGE molecule, resulting in intrahelical crosslinking, a ring structure (VIII) could be formed. Under acidic conditions (pH 4.5–6.0), amino groups were protonated to form products (IX). The degree of epoxy protonation and the reaction rate increased with a decrease in pH. The main reaction involved the attack of carboxyl nucleophiles of glutamic acid and aspartic acid at the end, with a larger steric hindrance of the protonated epoxy group to form ester bonds (X) and (XII). In addition, hydrolysis side reaction (X→XI) was observed under acidic conditions, similar to reaction III→V, and the hydrolysis rate under acidic conditions was faster than that under alkaline conditions, where more BDDGE could be consumed [123, 124].

**Fig. 6:**
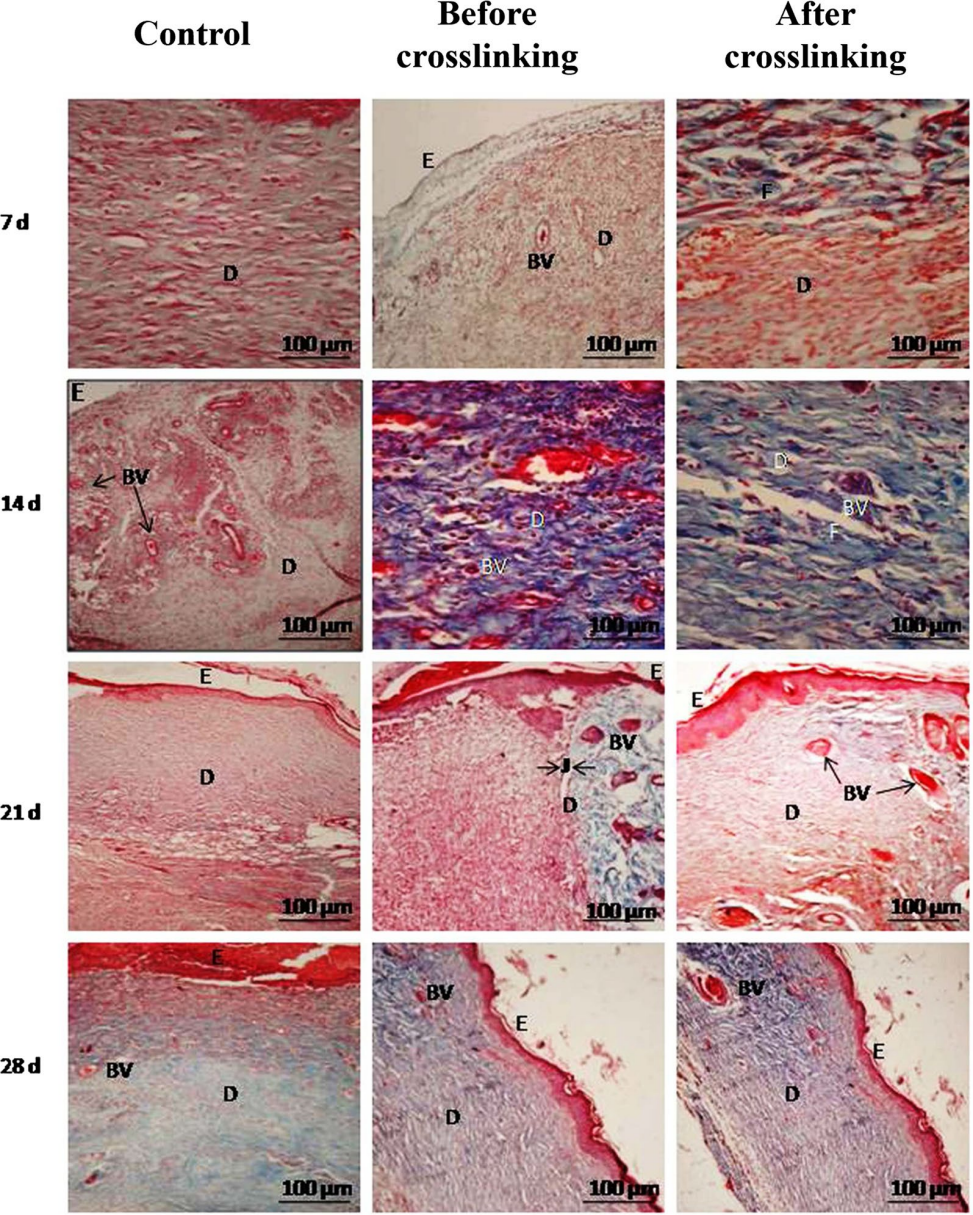
Representative histological images of wound sections taken after 7, 14, 21, and 28 days of Masson’s trichrome staining. Swim bladder tissue was implanted on to a skin surface wound in New Zealand white rabbits. BV, blood vessel; D, dermis; E, epithelial layer; F, fibroblasts. Reprinted with permission from Ref. [115]. Copyright 2015, Elsevier B.V

Kumar et al. crosslinked and fixed the swim bladder with BDDGE and found that the tensile resistance increased [114]. The BDDGE crosslinking group was lighter, white, and softer than the GA and self-crosslinking groups. Nonenzymatic degradation in an isotonic saline solution with 0.1% sodium azide or enzymatic degradation using collagenase Type I from *Clostridium histolyticum* in phosphate-buffered saline containing 0.2 mg/mL sodium azide showed that crosslinking treatment could significantly reduce the weight loss ratio [114]. Within the treatment duration of 12–72 h, the resistance to degradation increased with increasing reaction time which was better in the BDDGE group than that in the self-crosslinking group, but slightly worse than that in the GA group [114]. By monitoring the changes in free amino acid content, denaturation temperature, and water content in fixed tissue, the fixation rate could be determined, and the rate with BDDGE was greater than that of self-crosslinking, but less than that of GA crosslinking. Subsequently, the efficacy of the BDDGE crosslinked swim bladder as a skin wound dressing was further assessed in a full-thickness skin wound repair experiment in rabbits [115]. In comparison, before and after crosslinking, it was found that the regenerated fibers in the crosslinked group became smaller, thinner, and neatly arranged. Twenty-one days post-surgery, the number of neovascularization lesions was similar to that of normal skin. The total response of IgG in the serum of rabbits implanted with the BDDGE crosslinked biomaterial was lower than that in the fresh group, as assessed using enzyme-linked immunosorbent assay. Histologically, the crosslinked group showed improved epithelial cells, neovascularization, fibrous proliferation, and collagen fibers in an optimal arrangement as early as 21 days after transplantation (Fig. 6). Although the crosslinking effect of BDDGE on collagen is not as strong as that of GA, it can reduce the inflammatory reaction and has antibacterial activity [115]. Therefore, it has potential applications as a biological dressing in skin repair.

#### Self-crosslinking treatment

**Fig. 7:**
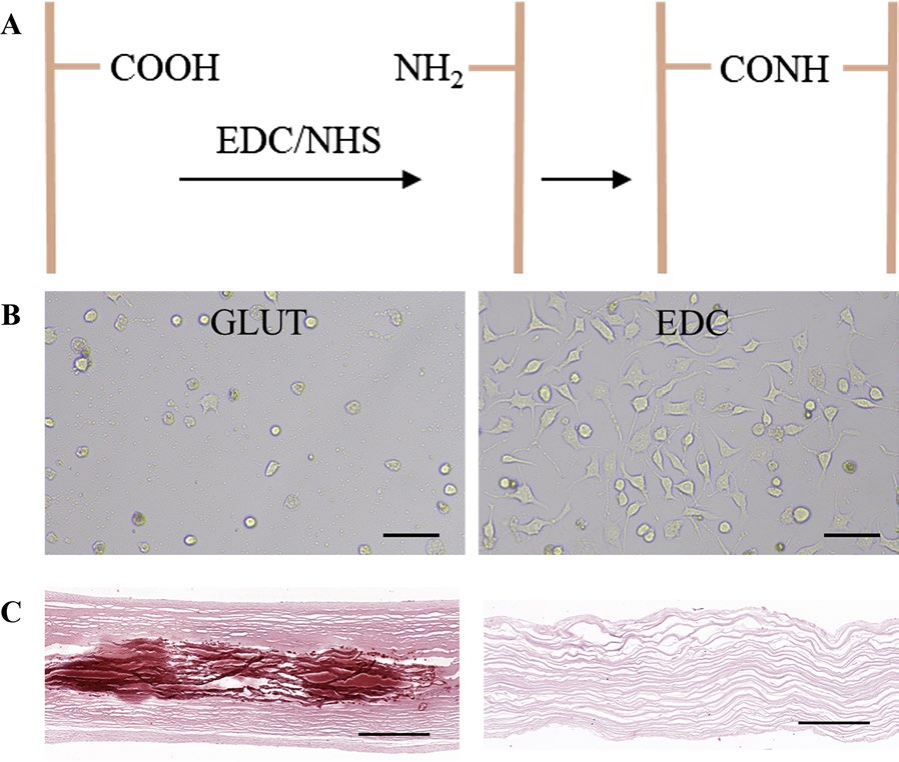
Schematic representation of the self-crosslinking mechanism with EDC/NHS (**A**). Morphology of L929 cells on crosslinked swim bladder with GA (GLUT) and self-crosslinked swim bladder with EDC/NHS (EDC) (**B**). Calcification of subcutaneous implants in rats (alizarin red staining, **C** scale bar = 100 mm. Reprinted with permission from Ref. [48]. Copyright 2021, IOP Publishing, Ltd

The self-crosslinking treatment of swim bladders catalyzed by EDC/NHS is different than that by bifunctional crosslinkers that bridge proteins through monomers or oligomers. The crosslinking reaction is completed by direct activation of the amino acid residues on the peptide chain to form amide bonds. Therefore, compared to GA crosslinking that may result in cytotoxicity caused by residual crosslinking agents, self-crosslinking has no such disadvantage [116]. Specifically, EDC catalyzes the reaction with the carboxyl residue of aspartic acid or glutamic acid on the protein to form an O-acyl isourea intermediate, which is unstable and reacts easily with the amino group on other amino acid residues to produce a crosslinked product connected by an amide bond (Fig. 7A) [48, 117]. Accompanying side reactions include the hydrolysis of intermediates back to a carboxyl or rearrangement to more stable N-acyl urea. In the crosslinking reaction, NHS is often added to form the EDC/NHS system because NHS can catalyze the reaction to form a more stable ester intermediate with an O-acyl isourea intermediate, leading to the inhibition of the other two side reactions, thereby improving the crosslinking yield [118, 119]. Totaro et al. found that the adjacent nucleophilic residues (lysine, arginine, histidine, or cysteine) had a negative impact on the results of the EDC/NHS crosslinking reaction. Furthermore, various amino acids, including methionine, tryptophan, and cysteine, display other side reactions with EDC/NHS [120]. Overall, EDC/NHS plays a catalytic role in crosslinking, which activates the carboxyl groups of collagen to react with the amino groups of collagen. The biggest advantage of self-crosslinking treatment with EDC/NHS is that no crosslinking residue is generated. Crosslinked porcine pericardium with EDC/NHS was implanted subcutaneously in rats, where strong cell infiltration and no cytotoxicity were observed [125]. Furthermore, the crosslinked group showed lower macrophage infiltration and higher collagen tissue integrity than the fresh group, indicating that the self-crosslinking treatment with EDC/NHS had better anti-immunogenicity. Moreover, neither the fresh group nor the crosslinked group showed calcification, indicating that the self-crosslinking treatment with EDC/NHS did not lead to an increased susceptibility to calcification.

In recent years, EDC/NHS has been employed for self-crosslinking swim bladder tissue that gives the material a loose and porous appearance when assessed by scanning electron microscopy (SEM) [48, 121]. Zhang et al. used EDC/NHS to self-crosslink swim bladder and found that after treatment, the strength and thermal deformation temperature of the material was increased, degradation rate was decreased, and physical and chemical properties of the material were markedly improved [121]. Unlike crosslinking with GA, the self-crosslinking treatment was non-toxic. Nonenzymatic and enzymatic degradation experiments in vitro showed that self-crosslinking treatment with EDC/NHS reduced the degradation rate, suggesting that the material would be less sensitive to degradation in vivo. The thermal denaturation temperature after self-crosslinking was also higher than that of the fresh material, which further confirmed that the stability of swim bladder-derived materials was improved. The cytotoxicity of mouse fibroblasts was measured by in vitro culture experiments, and there was no significant difference compared to that of the fresh material, confirming that self-crosslinking treatment with EDC/NHS was essentially noncytotoxic [114, 121]. Lan et al. also confirmed that swim bladder crosslinked with EDC/NHS was better than that crosslinked with GA, and the former was more conducive to the adhesion and growth of fibroblasts (Fig. 7B). In addition, the anti-calcification properties of the swim bladder crosslinked with EDC/NHS were significantly better than those of the swim bladder crosslinked with GA (Fig. 7C) [48].

Kumar et al. compared the physical properties of acellular swim bladder ECM crosslinked with GA, BDDGE, or EDC/NHS [114]. The protein content and free amino acids in the tissues treated with GA were the lowest, followed by those in the tissues treated with BDDGE and EDC/NHS. The degradation results were consistent with those for free proteins and free amino acids. The weight loss rate for the GA treatment was the lowest, followed by that for BDDGE and EDC/NHS. Therefore, GA had the best crosslinking effect on SBC, with the best stability of crosslinked collagen, followed by BDDGE, whereas EDC/NHS had the weakest crosslinking effect. However, compared with those treated with BDDGE and EDC/NHS, GA-treated tissues hardened, moisture content decreased, and tissue color changed.

## Applications of the swim bladder in biomedical field

The applications of swim bladder tissue in the biomedical field can be divided into direct and indirect. Direct application refers to the use of fish swim bladder tissue in the form of swim bladder-derived materials based on ECM after decellularization and crosslinking treatment, and as edible and pharmaceutical fish maw (dried swim bladder). Indirect application refers to the extraction of various useful ingredients from swim bladder tissue, mainly collagen peptides, collagen, gelatin, and their use in food, supplements, or other products.

### Direct applications

#### Soft tissue repair

Individuals experience various skin injuries in their daily lives. Wound healing can be treated using autologous, allogeneic, or xenogeneic skin transplantation or other wound dressings at the wound site. Autologous skin is the ideal substitute for damaged skin; however, this source is limited and large-scale skin repair is not possible. Allografts are mainly derived from donated organs. Organ donation is influenced by societal barriers and ideologies; moreover, graft rejection reactions limit the use of these sources. The use of mammal-derived xenotransplantation is also limited because of immune rejection reactions and risk of infectious diseases [126, 127].

An effective wound dressing is a material that can protect the wound, prevent infection, and promote repair. Wound dressings can be divided into two categories: synthetic and biological [49, 128]. Synthetic polymer dressings include polyethylene, polyvinyl alcohol, polytetrafluoroethylene, and siloxane elastomers, most of which are not biodegradable, and their mechanical properties are different from those of skin; hence, they are not ideal for practical applications [129, 130]. Biological dressings comprise collagen and non-collagen dressings (e.g., alginate, chitosan or other polysaccharides) [50]. Although alginate and chitosan have biological properties, such as hemostasis, anti-inflammatory, and antibacterial, their cytocompatibility and surface activity are not as good as those of collagen [131].

Collagen is an effective biomaterial for wound healing with strong hydrophilicity and good permeability. As a wound dressing, collagen has excellent biological characteristics, including biodegradability and non-cytotoxicity, and can promote directional cell adhesion, facilitate the proliferation and repair of epithelial cells, and promote wound healing [50, 132, 133]. The swim bladder contains a large amount of type I collagen, which has been shown to promote wound hemostasis through the regulation of coagulation factors and activation of exogenous coagulation, which assists in skin and mucosal injury repair [134, 135]. In addition, the process of wound healing is related to reactive oxygen species (ROS). Type I collagen can remove 2, 2-diphenyl-1-picrylhydrazyl radicals (DPPH•), superoxide anion radicals (O_2_-•), 2,2’-azino-bis-3-ethylbenzothiazoline-6-sulfonic acid radicals (ABTS•), and other free radicals, thereby acting as an antioxidant. Therefore, owing to its antioxidant properties, collagen derived from aquatic organisms is often used in skincare and wound healing products [136].

It has been shown that antigen epitopes on cells can cause tissue inflammation and immune rejection [137], and removing these epitopes can reduce or avoid adverse immune reactions in xenografts [13]. Bhanu et al. used both decellularized and fresh swim bladder for the repair of rabbit abdominal wall. The use of decellularized swim bladder resulted in less biochemical changes in the blood, less tissue reaction, lower antigenicity, and better tissue repair than when fresh swim bladder was used [108, 138]. This was mainly because of the formation of a loose fibrous layer and a three-dimensional porous structure in the decellularized swim bladder matrix. Another study has confirmed that the three-dimensional reticular structure facilitates cell migration and proliferation and can accelerate the process of wound repair [47]. Baldursson et al. further confirmed that the effect of swim bladder matrix on the wound healing speed may be better than that of decellularized porcine skin and cow leather products [139]. Based on these studies, Jalali et al. first fabricated an acellular fish swim bladder (AFSBM), loaded it with exogenous HA as a carrier, and tested its wound repair ability using the rat back trauma model [46]. Macroscopic and histological assessments of wound healing revealed that, compared with that of the other groups, the wound area of the AFSBM-HA group decreased rapidly, indicating faster wound healing. On the seventh day after the injury, the process of epidermal formation and angiogenesis in the AFSBM group was more advanced than that in the control group. Furthermore, the density of inflammatory cells decreased significantly in the AFSBM-HA group, and the number of fibroblasts and collagen production increased significantly [46].

**Fig. 8:**
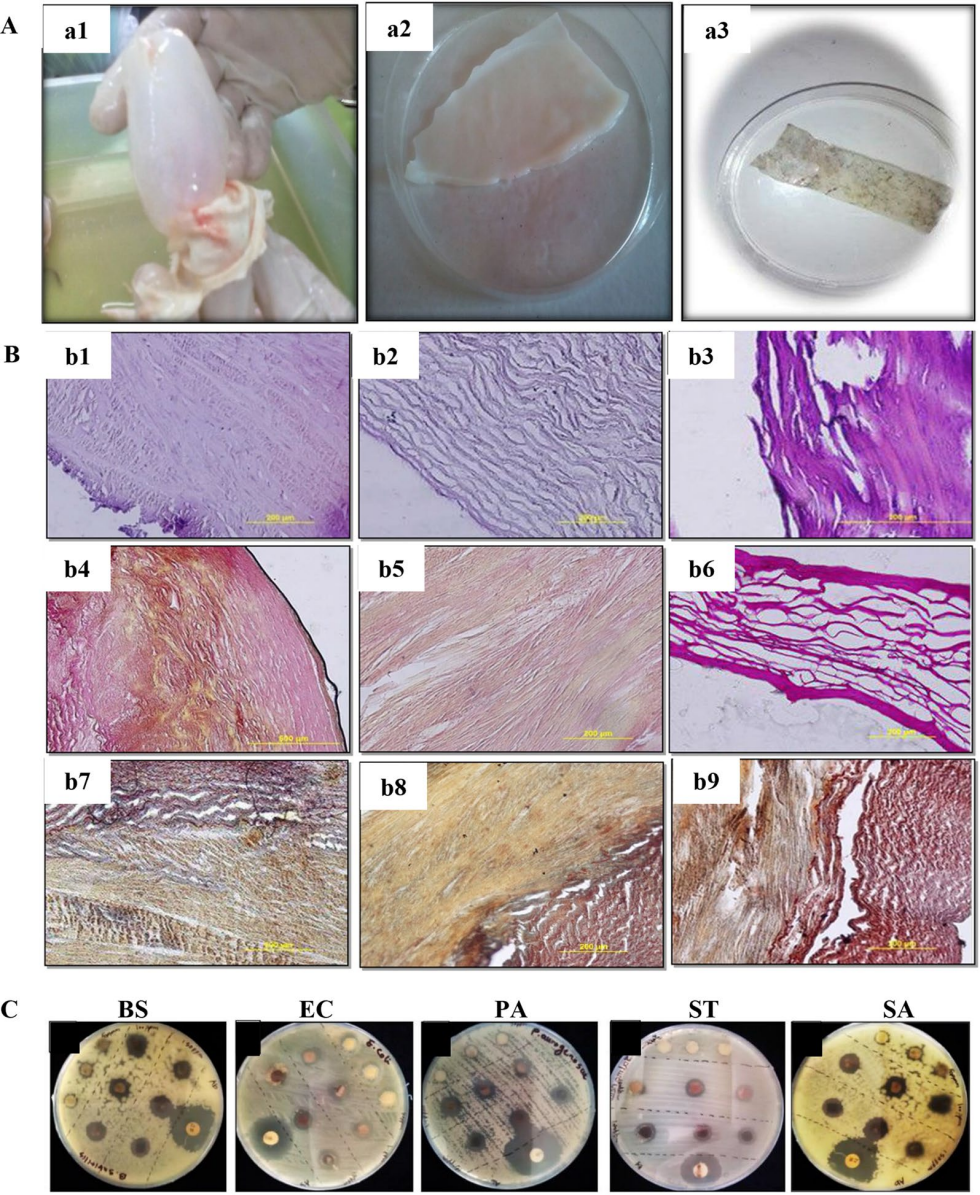
Experimental results of acellular swim bladder-loaded AgNPs: Optical photographs of swim bladder before and after decellularization and impregnation with AgNPs (**A**), where a1, a2, and a3 represent fresh swim bladder, decellularized, and AgNP-soaked samples, respectively; histological staining analysis of fresh swim bladder before (b1, b4, and b7) and after decellularization with 0.5% SDS (b2, b5, and b8) and 1.0% SDS (b3, b6, and b9) (**B**), among which b1–b3, b4–b6, and b7–b9 are H&E, picro-fuchsin, and orcein-picroindigocarmine staining, respectively; bacterial experiment results (**C**), in which BS, EC, PA, ST and SA represent Bacillus subtilis, Escherichia coli, Pseudomonas aeruginosa, Salmonella typhimurium and Staphylococcus aureus, respectively. Reprinted with permission from Ref. [27]. Copyright 2020, Elsevier B.V

Howaili et al. developed a novel antimicrobial wound dressing based on a swim bladder doped with silver nanoparticles (AgNPs) [27]. Optical photographs of the swim bladder matrix before decellularization, after decellularization, and doped with AgNPs are shown in Fig. 8A, and the histological results are shown in Fig. 8B. When the swim bladder was decellularized by 3 min of freeze-thawing and soaking in liquid nitrogen with chemical decellularization using 0.05% SDS, the ECM component was retained without any cellular or nuclear material observed [27]. Moreover, the results of H&E, picro-fuchsin, and orcein-picroindigocarmine staining confirmed that the bioscaffold derived from the swim bladder consisted of a large amount of collagen, elastin fibers, and muscles. The antibacterial properties of the scaffolds were evaluated by determining their antimicrobial activity based on disc diffusion and growth inhibition methods against several common bacteria. The results (Fig. 8C) indicated significant antibacterial activity [27]. The above studies confirmed that the swim bladder matrix not only possesses good wound healing qualities but can also be used as a carrier to load various material components conducive to wound healing and repair.

However, some researchers have proposed that decellularized treatment greatly reduces the antigenicity of the tissue, but does not eliminate it. Hence, the direct use of decellularized tissue in xenotransplantation may cause cross-species responses to structural proteins [140]. Crosslinking can further reduce this response [141, 142]. Kumar et al. used BDDGE to crosslink the decellularized swim bladder matrix to explore the effect of crosslinking treatment on wound healing [115]. Compared to those in the fresh group, the total immunoglobulin serum reaction and peripheral blood lymphocyte stimulation index of rabbit blood in the crosslinked group decreased significantly. Thus, BDDGE crosslinking can significantly reduce the degree of inflammatory reaction in the swim bladder matrix, stimulate angiogenesis, and produce antibacterial activity. Therefore, crosslinking can further improve the biocompatibility of the swim bladder matrix and promote the repair of skin wounds.

#### Dural repair

Dural rupture is a common complication in neurosurgery and spinal surgery [143]. Dural repair can effectively prevent postoperative complications such as cerebrospinal fluid leakage, meningitis, and spinal canal or intracranial infection [144, 145]. Depending on their source, the materials used for dural repair can be divided into four categories: autologous tissue, allogeneic tissue, xenogeneic materials, and synthetic materials. Autologous tissues have no immunogenicity; however, the size and shape of the materials are limited, and there is a risk of additional surgical injury to patients [146, 147]. Allogeneic tissues have been gradually withdrawn because of the risk of prions and other infectious diseases [148, 149].

According to statistics, the most widely used dural repair materials worldwide are xenogeneic materials [150–153]. Xenogeneic biomaterials generally use the basic structure of fiber scaffolds in ECM, as from porcine small intestine submucosa, animal pericardium, and animal dermis, to make repair materials through freeze-drying, crosslinking, or acellular treatment. In the 1990s, the Food and Drug Administration in the United States approved GA-treated bovine pericardium as a dural repair material [154]. GA crosslinking treatment changes the biological properties of the raw materials and improves their safety for clinical use. However, it introduces toxic aldehyde groups while removing heterologous protein antigens that cause inflammatory reactions, and the material has poor mechanical properties and a rapid degradation rate [155]. Moreover, xenogeneic biomaterials from mammals may pose risks, such as the carrying of Creutzfeldt Jakob disease, which has a very high mortality rate. Therefore, direct implantation of xenogeneic repair materials into the human nervous system is a cause for concern [156].

Unlike pathogenic bacteria in mammals, pathogens in fish are almost never transmitted to humans [157]. Therefore, the materials extracted from fish are safer than those extracted from mammals. Additionally, fish do not contain many immunogens such as α-Gal; therefore, the biomaterial obtained from fish has lower immunogenicity [158]. Thus, fish-derived materials have been developed to prepare dural repair materials, including fish skins and swim bladders. Among these, swim bladders are easy to obtain, widely sourced, and simple to handle; hence, they have potential applications as dural repair materials.

**Fig. 9:**
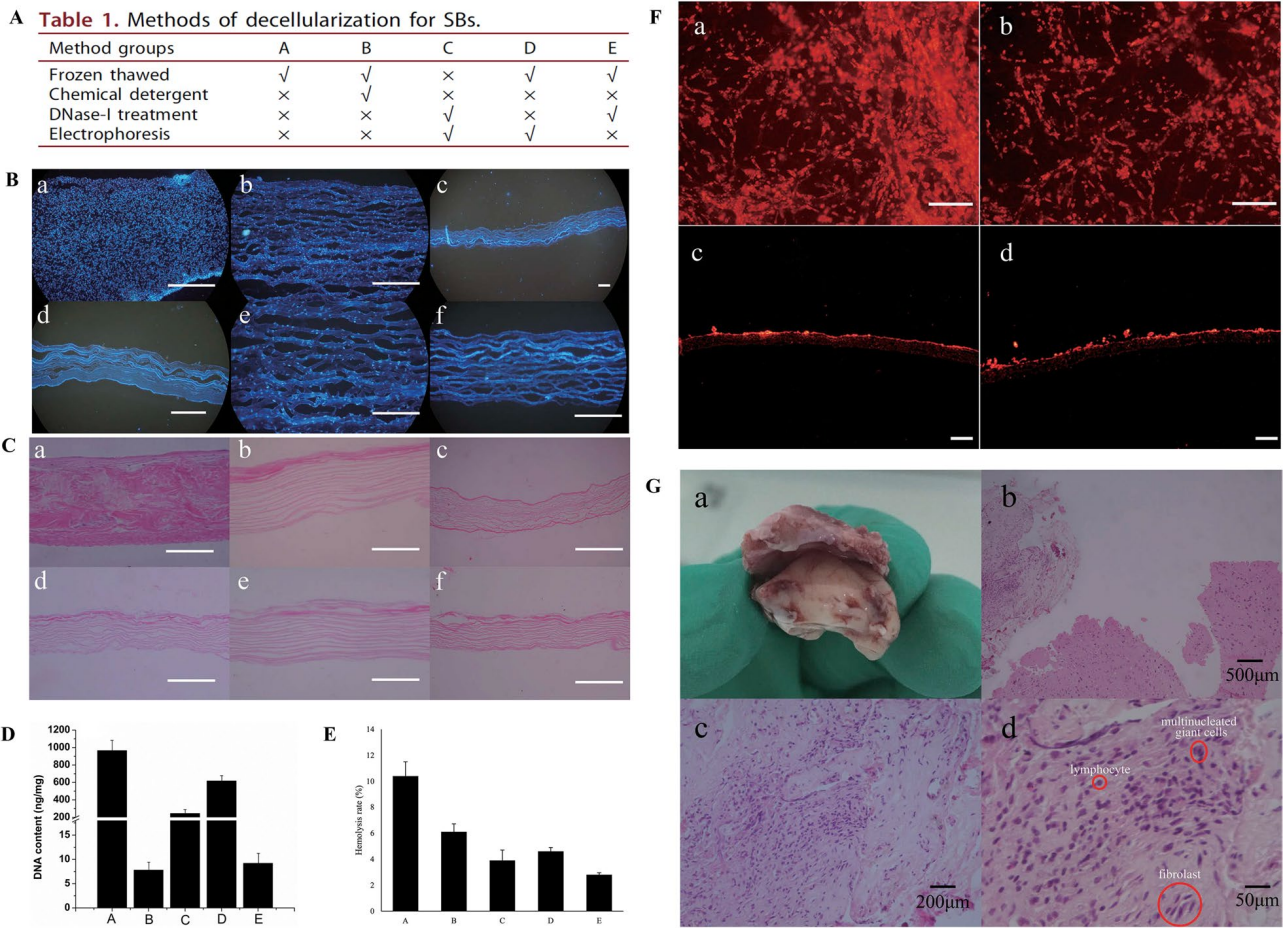
Swim bladder tissue treated using different decellularized methods: Details of different decellularized treatment methods (A); DAPI staining before and after decellularized treatment (**B**), where (a) is fresh swim bladder, and (b–f) correspond to groups A–E in (**A**), respectively; the physical structure of the tissue was observed using H&E staining before and after decellularized treatment (**C**); DNA test results after decellularized treatment (**D**); hemolysis rate after decellularized treatment (**E**); in vitro co-culture assay with mouse embryonic fibroblasts (MEFs) (**F**), in which (a, c) and (b, d) are groups E and B in image A; immunogenic assessment of three months after dural repair (**G**), in which a is the overall appearance of the excised material and b-d show the fibroblast distribution and inflammation at the dural repair site. Reprinted with permission from Ref. [47]. Copyright 2019, Taylor & Francis

The decellularized swim bladder matrix forms a loose fibrous layer with a three-dimensional porous structure. Fibroblasts can adhere, migrate, and proliferate on the surface or inside of the matrix, gradually completing the repair and regeneration of the dura mater, and will not cause brain adhesion or serious inflammatory reactions [159]. The acellular swim bladder matrix exhibits good biocompatibility and may be highly suitable as a dural substitute. Li et al. used freeze–thaw, chemical detergent, DNase-I enzymatic hydrolysis, and electrophoresis methods to decellularize the swim bladder [47]. On examination of its physical structure, residual DNA content, mechanical properties, and effect on hemolysis rate, the biocompatibility of decellularized swim bladders was further evaluated by co-culture of mouse embryonic fibroblasts (MEFs) in vitro and dural repair surgery [47]. It was found that the material prepared by the combined treatment of freeze–thaw and DNase-I was the most suitable substitute for the dura mater (Fig. 9A). The material was thoroughly decellularized using these two methods (Fig. 9B). After decellularization, a loose fibrous layer and a three-dimensional porous structure were formed (Fig. 9C). The residual DNA content was low (9.2 ± 2.0 ng/mg) (Fig. 9D). The mechanical properties met the clinical requirements (the maximum tensile and suture tear strengths were 34.77 ± 4.28 N and 7.15 ± 1.84 N, respectively), and the hemolysis rate was 2.8 ± 0.15% (Fig. 9E). In the co-culture experiment with MEFs, decellularized swim bladders supported cell adhesion, migration, and proliferation (Fig. 9F). Three months after dural repair, the mice were killed and their brains were removed and examined. It was found that most decellularized swim bladders were replaced by connective tissue that did not adhere to the cerebral cortex or skull. Many fibroblasts grew into decellularized swim bladders. There was no obvious fibrous capsule and the inflammatory reaction was mild. Only a small number of multinucleated giant cells and lymphoid cell infiltrates were observed (Fig. 9G). These results confirmed that the swim bladder tissue decellularized by the combination of freeze–thaw and DNase-I treatment can be used as a dural patch. Animal experiments have confirmed that it can effectively prevent cerebrospinal fluid leakage and is gradually replaced by autologous connective tissue. This new substitute improves the repair and regeneration of the dura mater without causing adhesion or severe inflammation [47].

However, the fiber structure may be damaged to different degrees by different decellularization methods; therefore, it is necessary to comprehensively select the decellularization method to be used based on the differing mechanical properties inherent to swim bladders sourced from different fish species and also on the size of the dural area being repaired. Crosslinking is another strategy for enhancing mechanical strength. Owing to the increase in the stiffness of biological tissue treated with GA, the mechanical strength can be improved to a certain extent [160]. Zhang et al. crosslinked the swim bladder matrix with 0.3% GA, and its mechanical and physical properties were similar to the dura mater of normal adults [87]. Dural repair assessed in rabbits showed healthy wound healing, without complications, such as cerebrospinal fluid leakage or subcutaneous effusion, and the graft did not degenerate and calcify [87].

#### Cardiovascular repair

Currently, cardiovascular materials such as vascular grafts, bioprosthetic heart valves, and cardiovascular stents used in clinic are prone to calcification and mechanical failure [12, 161–164]. The swim bladder is rich in collagen and elastin, and its composition is similar to that of traditional bovine pericardium valve materials [12]; the latter, however, has greater hardness and is more easily damaged by folding [163, 164]. In contrast, the ductility of swim bladder-derived biomaterial is better in both the axial and circumferential directions. Better ductility makes swim bladder-derived biomaterial less prone to damage during folding, which is more conducive to applications in the cardiovascular field, thereby minimizing invasive interventions from the perspective of mechanical properties [48, 165]. Therefore, researchers are exploring the feasibility of sourcing biomaterials from swim bladder for cardiovascular applications.

**Fig. 10:**
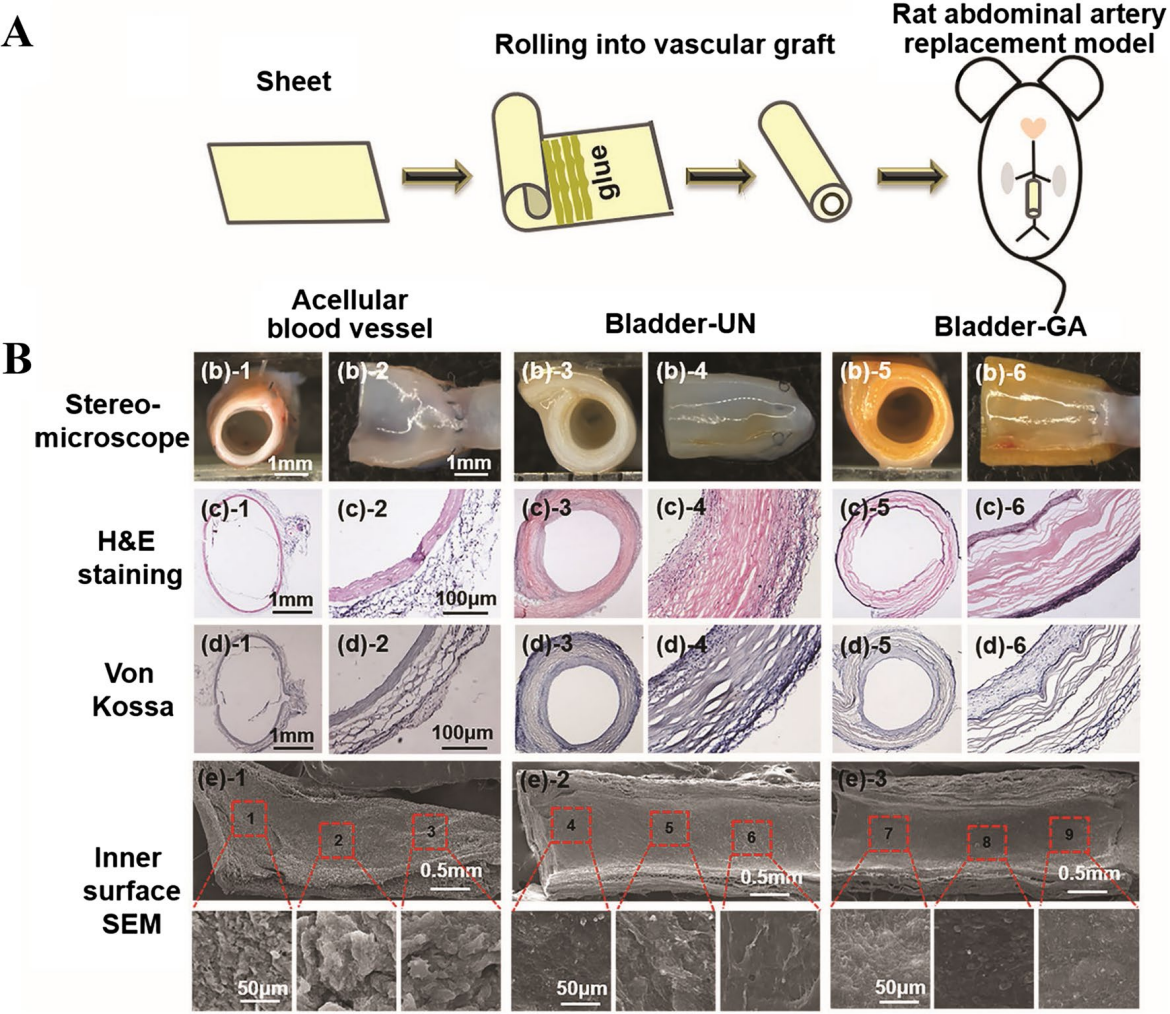
Schematic diagram of the preparation of swim bladder-derived small-diameter vascular grafts by the rolling process (**A**) and the histological staining and morphological observation results of the extracts after celiac artery replacement in rats (**B**). Reprinted with permission from Ref. [12]. Copyright 2019, Wiley–VCH GmbH

Liu et al. first introduced a natural material extracted from swim bladder, which was prepared by decellularization and GA crosslinking [12]. The swim bladder was particularly rich in elastin content, and had a higher elastic modulus than bovine pericardium. Additionally, the degree of calcification of this material was significantly lower than that of bovine pericardium when tested in in vitro calcification and in vivo experiments using a subcutaneous implantation model in rats. Furthermore, in vitro experiments demonstrated good cytocompatibility, hemocompatibility, and resistance to degradation by enzyme. On this basis, a small-caliber vascular graft was developed using this material through a rolling method for an in situ implantation experiment in a rat celiac artery replacement model (Fig. 10A). The experiment confirmed that this artificial small vessel showed good performance, exhibiting in high permeability and low calcification, verifying the feasibility and advantages of swim bladder-derived materials in the field of cardiovascular grafts (Fig. 10B). The material showed better anti-calcification properties than bovine pericardial materials, appropriate mechanical strength and stability, and good hemocompatibility and cytocompatibility [12]. These observations indicate that swim bladder will become an ideal candidate from which to source biomaterials for the cardiovascular applications.

Subsequently, Bai et al. further extended this work [166], where they first decellularized the swim bladder of *Carassius auratus,* coated it with rapamycin, and made the material into massive patches or tubular grafts, which were implanted into the rat vasculature. Rapamycin-coated patches resulted in decreased intimal thickness in aortic and inferior vena cava patch-plasty models. After the rapamycin-coated swim bladder-derived vascular grafts were implanted into rat aorta, neointima and macrophage numbers decreased compared to those in implants without rapamycin coating [166]. These data further verified the feasibility and safety of swim bladders as tissue-engineered vascular patches or grafts.

**Fig. 11:**
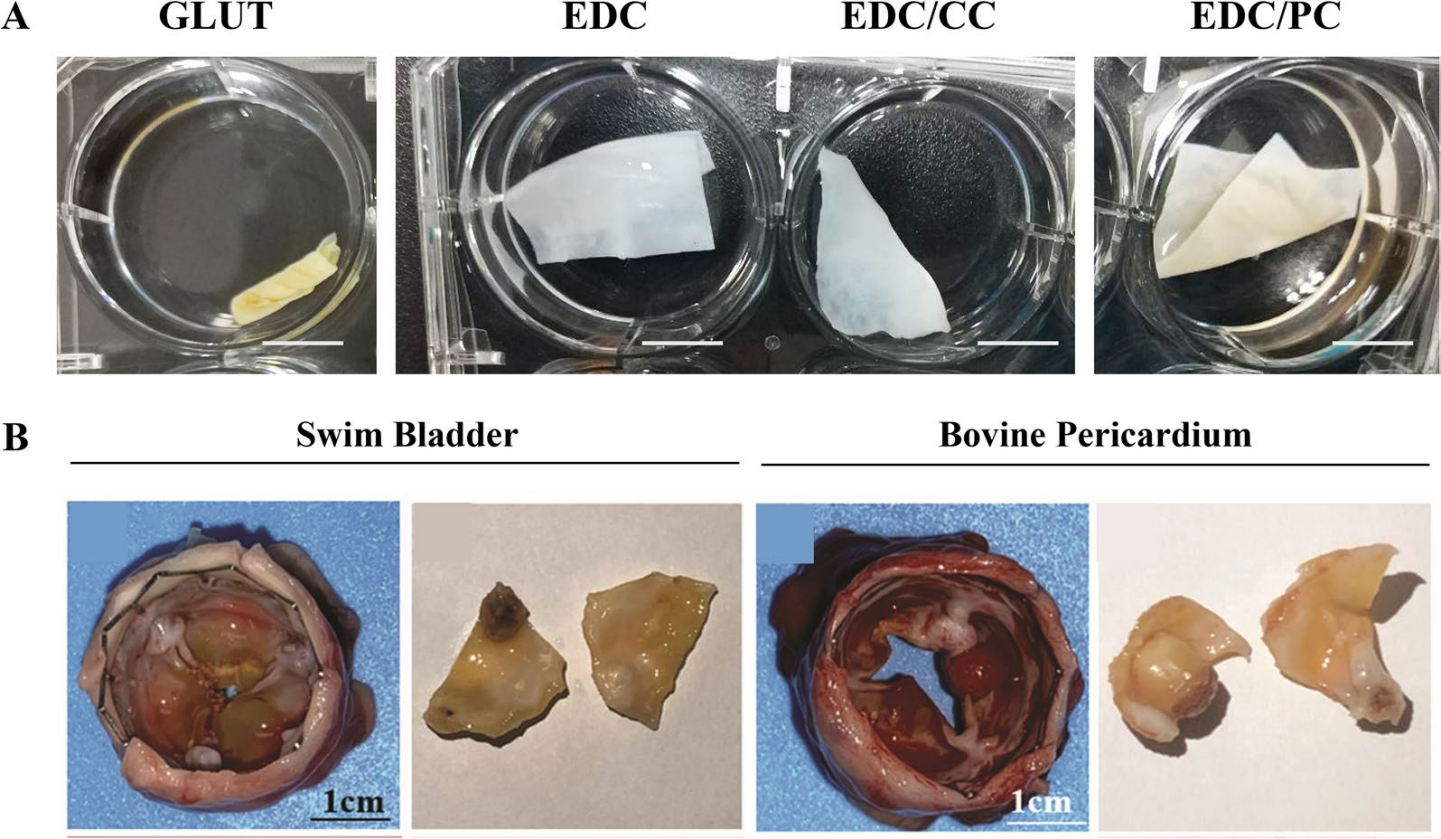
"Dry membrane" material (**A**) made of swim bladder-derived biomaterial. Reprinted with permission from Ref. [48]. Copyright 2021, IOP Publishing, Ltd. Overall appearance (**B**) of the pulmonary valves implanted in sheep. Reprinted with permission from Ref. [85]. Copyright 2021, Royal Society of Chemistry

In addition to being used as vascular grafts or patches, swim bladder-derived biomaterials after crosslinking treatment could be used as bioprosthetic heart valves. For example, Lan et al. successfully developed a "dry membrane" from swim bladder-derived biomaterial by using different crosslinking methods [48]. At present, minimally invasive interventional valve surgery in clinic mostly uses GA-crosslinked "wet membrane," which not only shows aldehyde residue toxicity but is also not conducive to storage and transportation. When used, the wet membrane products require on-site assembly by doctors, delaying the surgical procedure and increasing risk. As a solution, the development of "dry membrane" products that can be preassembled has been proposed [48]. Here, the porcine pericardial material is GA-crosslinked and treated using a unique "dry membrane" treatment technology to obtain "dry membrane" and related valve products that can be repeatedly pre-pressed and pre-folded [167]. However, it was found that the "dry membrane" material from the porcine pericardium could not be flattened quickly after being pre-pressed and pre-folded in water. Therefore, the use of swim bladder-derived biological tissue was proposed. It was found that compared with those treated with GA, the materials treated with EDC could flatten more quickly in water (Fig. 11A), which may be because of the higher degree of crosslinking and tissue hardening with GA treatment compared to that with EDC [114, 160]. Recently, Li et al. successfully developed a swim bladder-derived pulmonary valve by stitching GA-crosslinked swim bladder-derived material onto a cobalt chromium alloy stent [85]. In vitro experiments showed that the crosslinked carp swim bladder exhibited better biocompatibility and anti-calcification performance than the bovine pericardium. Its durability was verified by an in vitro fatigue test (Fig. 11B). The feasibility of the crosslinked swim bladder tissue, sutured to a cobalt chromium alloy stent to create a pulmonary valve, was verified by subcutaneous implantation in rats [85]. Subsequently, the material was tested in situ, as a pulmonary valve replacement in sheep, which confirmed its excellent anti-calcification, immunocompatibility, endothelialization, and hemodynamic properties [85], suggesting that swim bladder-derived biomaterial may be used for bioprosthetic heart valves.

However, compared with other cardiovascular materials, swim bladder-derived cardiovascular materials are thicker because of their rich collagen and polysaccharide components. Hence, meeting the thickness requirements of small-diameter vascular grafts or bioprosthetic valves using minimally invasive interventions is difficult. Therefore, the effective regulation of the thickness of swim bladder-derived cardiovascular materials, such as by selecting suitable fish species or methods and processes of enzymatic hydrolysis and crosslinking, should be considered in future research.

#### Edible and pharmaceutical fish maw (dried swim bladder)

The swim bladder contains a large amount of collagen, which is an important raw material that is easily absorbed as a supplement protein in the human body, helping to improve the nutritional status and metabolism of tissues [86, 168, 169]. Fish maw, which is a dried product of swim bladder and well-known alongside bird’s nest and shark’s fin, is one of the “Four Treasures of the Sea” and has a long history as an ideal food therapy product [170–172]. Wen et al. [172, 173] and Guo et al. [168] systematically evaluated the main components of several fish maws including those from *Cynoscion acoupa*, *Aggregatox talabonoides*, *Sciades props*, *Protonibea diacanthus*, *Lates niloticus*, and *Nibea coibor*. The results showed that swim bladders were rich in protein and functional amino acids, with a low fat content, making them ideal high-protein and low-fat foods. For example, *Cynoscion acoupa* maw contains many polyunsaturated fatty acids including arachidonic acid, docosahexaenoic acid, and eicosadienoic acid, which results in a high hypocholesterolemic/hypercholesterolemic ratio that is beneficial for preventing atherosclerosis and thrombosis. In addition, Dai et al. showed that peptides extracted from swim bladders can help treat ulcerative colitis [174]. According to the practitioners of the Traditional Chinese medicine, the swim bladder has a sweet and mild taste and has functions such as nourishing Yin and blood, stopping bleeding, tonifying the kidney, and consolidating essence.

The preparation of fish maw involves the removal of the swim bladder from the fish, stripping of the blood film, cleaning, and fully drying in the sun; alternatively, the swim bladder is soaked with a certain concentration of alum to remove the slime, cut with scissors to form a sheet, washed, and partially dehydrated, then flattened and dried completely in the sun [171, 175]. Fish maws usually need to be soaked and rehydrated prior to cooking. Based on market research, we summarized the species, photographs of the product (from the Internet), regions of origin, characteristics and advantages, and medicinal effects of typical fish maw products (Table 2).

**Table 2:** Summary of the market research of typical fish maw products

Species	Photographs	Regions of origin	Characteristics and advantages	Medicinal effects
*Barramundi*		Australia, China, India, and countries in Southeast Asia (mainly)	It is shaped like a capital Y; large-scale production; cheap; high nutritional value; good collagen quality	Regulates secretion and enhances immunity
*Nile perch* *(Lates niloticus)*		Kenya, Tanzania, Uganda, and other countries in the Nile Valley freshwater regions	Large-scale production; cheap; high nutritional value; good collagen quality; thick meat	Maintains beauty and has antiaging properties
*New Zealand ling*		New Zealand (mainly) and Europe	Contains a large amount of deep-sea minerals and collagen components. Large-scale production; moderate price	Moisturizes skin and removes wrinkles
*Suriname ling*		Argentina, Brazil, Ecuador, Guyana, Peru, Suriname (mainly), Uruguay, Venezuela, and other countries	It has two "ears" on its head, a tail, and saturated flesh. large-scale production; moderate price	Regulates secretion and enhances immunity
*Totoaba macdonaldi*		Mexico	The maw has two long whiskers that are nodular in shape; scarce; expensive; only for collection	Good hemostatic effect
*Bahaba*		China	It has been listed as a national second-class protected animal and is currently endangered; scarce; expensive; only for collection	Good hemostatic effect
*Red billed* (e.g., *Double spined yellow croaker, Brown catfish, and Farmed catfish*)		Indian Ocean (mainly), Nan'ao Island in China, Southeast Asia (mainly), and Western North Pacific	Scarce in the wild; expensive; the gelatin is delicate and has good viscosity; after stewing, the soup is clear	Aids in postoperative recovery and regulates secretion
*Otolithoides biauritus*		India, Peru, South Australia Island of China, and Vietnam	Large-scale; moderate price	Cough relief, moistening of the airways in lung, tonifying kidneys, and strengthening body’s essence
*Perciformes totoaba*		Indonesia, Laos, Philippines, Southeastern waters of China, Thailand (mainly), and Vietnam	Medium quality, with a strong fishy odor; varying prices	Nourishes blood, promotes blood circulation, enhances immunity, and promotes wound healing
*Brotula clarkae*		Pacific Ocean from the southern Gulf of California to Colombia	It is white and shaped like a "butterfly". The collagen content is relatively high	Nourishes the skin, removes wrinkles, and replenishes the blood to promote blood circulation
*Boesemania microlepis*		Indonesia, Malaysia, Thailand, and Vietnam (mainly)	The shape of the unopened fish maw is like a hammer; it is accompanied by 2–6 short and small whiskers	Helpful for cough, asthma, and bronchitis
*Eleutheronema tetradactylum*		Bangladesh, China, India, Indonesia, Myanmar, and Thailand	Large-scale production and moderate price; the gelatin is slightly soft, and the soup is slightly viscous	Maintains beauty and has antiaging properties
*Conger-pike eel*		Central and South America	It is a long, narrow, and cylindrical; large-scale production and moderate price	Maintains beauty and has antiaging properties

However, the preparation required before eating fish maw involves multiple steps, which are bothersome and time-consuming, and results in some loss of nutrients. Moreover, the absorption rate of the collagen in fish maws is low, which affects its efficacy. Therefore, many studies have focused on the indirect application of swim bladders based on extracted collagen peptides (smaller molecular weights) and collagen and gelatin (higher molecular weights).

### Indirect applications

#### Fish collagen peptide extraction

In recent years, some researchers have attempted to extract collagen peptides with smaller molecular weights from swim bladders using enzymatic hydrolysis techniques [174]. These small molecule active peptides are more easily absorbed and utilized, enhancing their efficacy while also increasing their convenience of consumption, meeting the needs of consumers for nutritional health, convenience, and fast-food characteristics. Li et al. performed enzymatic hydrolysis on the swim bladder of the Atlantic cod (*Gadus morhua*) and extracted two bioactive peptides, SWP-I and SWP-II, with molecular weights of 4,976 and 1,960 Da, respectively [72]. SWP-I and SWP-II effectively scavenged DPPH•, hydroxyl radicals (HO•), and O_2_-•, and exhibited high Fe^2+^-chelating activity. At the same concentration, the scavenging ability of SWP-II was higher than that of SWP-I. Cytoprotective experiments also showed that SWP-I and SWP-II possessed ROS scavenging properties. In a hydrogen peroxide (H_2_O_2_)-induced premature senescent model using human fetal lung diploid fibroblast cells, pretreatment with SWP-I and SWP-II improved the cell survival rate and inhibited SA-β-Gal activity and apoptosis [72]. In conclusion, polypeptides extracted from Atlantic cod have excellent antioxidant and antiaging properties.

Cai et al. identified various antioxidant pentapeptides such as FPYLRH, FYKWP, and FTGMD from the hydrolysates of *Miiuy croaker* swim bladder, and further investigated the protective effects of these antioxidant peptides on human umbilical vein endothelial cells (HUVECs) using an H_2_O_2_-induced oxidative damage model [176]. When 100 mg/mL FPYLRH was added, the viability of HUVECs with H_2_O_2_-induced oxidative damage increased. FPYLRH significantly increased the levels of superoxide dismutase and glutathione peroxidase and decreased the levels of ROS, malondialdehyde, and nitric oxide [176]. This study confirmed that FPYLRH significantly reduced H_2_O_2_-induced stress in HUVECs and could be used as a potential natural antioxidant in the functional food industry. Zheng et al. used five different proteases to hydrolyze the *Nibea japonicar* swim bladder and found that the hydrolysate prepared with neutrase containing collagen peptides had the greatest effect on scavenging DPPH•, with a maximum DPPH• clearance rate of 95.44% [177]. Furthermore, collagen peptides with a molecular weight of less than 1 kDa were obtained through ultrafiltration, and were found to have good scavenging activity against HO•, ABTS•, and O_2_-•. Moreover, these peptides significantly promoted the proliferation of HUVECs and reduced H_2_O_2_-induced oxidative stress damage in HUVECs [177].

However, there are few extraction strategies to obtain collagen peptides with smaller molecular weights from swim bladders, and the potential products are expensive. Owing to their ability to provide high-quality collagen supplementation and antioxidant activity, swim bladder-derived collagen peptides are suitable for individuals with weak immune systems and related health conditions, as well as for female consumers that use collagen products for beauty, skincare, and antiaging purposes. Therefore, with the continuous improvement of extraction technologies, an increasing number of swim bladder-derived collagen peptide products can be obtained, with a broader range of uses including applications as constituents in functional foods, health foods, cosmetics, beauty and antiaging products, immune-enhancing products, and nutritional supplements.

#### Fish collagen and gelatin extraction

Compared with the extraction of collagen peptides with smaller molecular weights, the extraction of collagen and gelatin (having higher molecular weights) has been researched earlier and is a more mature technology. Collagen and gelatin are usually separated from the by-products of terrestrial animals (such as cattle, pigs, and poultry). Owing to its excellent biocompatibility, biodegradability, and weak antigenicity, collagen is widely used in food, pharmaceutical, and cosmetic industries [178]. However, outbreaks of BSE, transmissible spongiform encephalopathy, FMD, and avian influenza have caused anxiety among consumers who consume collagen and collagen-derived products sourced from these terrestrial animals [31]. Moreover, some religious and ethnic groups, such as Jews and Muslims, cannot accept collagen from pigs and other animals not slaughtered ritually [179]. Therefore, the global demand for collagen from alternative mammalian sources such as aquatic animals is increasing. With the rapid development of the Chinese fish processing industry, a large number of by-products have been produced, accounting for 50–70% of the raw materials [180]. Therefore, optimizing the use of these by-products is a promising way to protect the environment, produce value-added products, increase the income of fish processors, and create new employment and business opportunities.

Swim bladders, with or without decellularization, are mainly used as a source of collagen to form hydrogels or biological adhesives. In the preparation process, the swim bladder is usually cut into small pieces and can be further smashed using mechanical forces, such as stirring and pounding, to facilitate the collagen extraction [36, 181]. Acetic acid solvent extraction is often used to extract collagen from swim bladder [28, 31, 65]. As early as 2012, Liu et al. reported the difference in extracting pepsin soluble collagen from different parts of bighead carp, such as the fin, scale, skin, bone, and swim bladder, using acetic acid [31]. Pepsin soluble collagen extracted from internal tissues (swim bladder and fish bone) had slightly higher thermal stability than those extracted from external tissues (fins, scales, and skin) [31]. All of the pepsin soluble collagen were soluble at an acidic pH (1–6), but their solubility decreased when the NaCl concentration exceeded 30 g/L. It was pointed out that pepsin soluble collagen extracted from these five tissues can be used as a potential substitute for mammalian collagen [31]. The general process of extracting collagen from the swim bladder involved soaking a certain amount of swim bladder in 0.1 M NaOH for 36 h and replacing the alkaline solution every 12 h. In this step, the sample/alkaline solution ratio was 1:30 (w/v) to ensure effective mixing, and the sample/butanol solution ratio was 1:30 (w/v) in the subsequent degreasing treatment. For the latter step, sample was suspended in 10% (v/v) butanol for 36 h, and the solution was changed every 12 h. The degreased substance was thoroughly washed with cold distilled water and then suspended in 0.5 M acetic acid containing 0.1% (w/v) pepsin for 3 d. The viscosity of the solution increased significantly during the extraction process. A sample liquid ratio of 1:40 (w/v) was used to ensure that the solution maintained an appropriate viscosity and could be magnetically stirred. The final solution was dialyzed against cold distilled water, and pepsin soluble collagen was obtained after freeze-drying [31]. Subsequently, Zhang et al. conducted similar studies using sturgeons [182]. The study found that the extraction of collagen in the swim bladder was the highest, up to 18.1% (collagen dry weight/tissue wet weight), compared to that from other issues. The yield of collagen from the skin was 11.9% and was very low from scales, muscle, digestive tract, notochord, and nasopharynx cartilage, at 2.1%, 0.4%, 0.4%, 0.8%, and 0.03%, respectively [182]. Through SDS-PAGE and amino acid composition analysis, the collagen from scales, skin, muscle, swim bladder, and digestive tract was identified as type I, and the collagen from the notochord and nasal cartilage was identified as type II [182]. In terms of fiber formation, the turbidity of the swim bladder and skin collagen increased faster than that of porcine tendon type I collagen. The time to reach maximum turbidity was shorter, and the fibers formed were thicker.

Gelatin is the product of the partial hydrolysis of collagen under acidic, alkaline, enzymatic, or high-temperature conditions [181]. It is also homologous to collagen. Collagen has a rod-shaped triple-helical structure [183], which breaks when it hydrolyzes to gelatin; hence, despite its amino acid composition being similar to that of collagen, it lacks the biological activity of collagen [184]. Gelatin can be further hydrolyzed into collagen peptides. Gelatin is soluble in hot water and has excellent physical properties such as gel strength, affinity, high dispersion, low viscosity, dispersion stability, and toughness. It is widely used in modern manufacturing, particularly in processed foods, pharmaceuticals, cosmetics, and photographic products [185]. Based on its usage, gelatin can be divided into medicinal gelatin, edible gelatin, photographic gelatin, and industrial gelatin. Medicinal gelatin is mainly used in the manufacture of soft and hard capsules, tablet icing, wound dressings, hemostatic sponges, and tissue engineering scaffolds. Edible gelatin can be used in meat jelly, food additives, cans, candy, ice cream, ham sausage, skin jelly, soda suspension agents, and starch agents. Photographic gelatin is mainly used for photocopying, whereas industrial gelatin is used in plywood, gauze, sandstone, and adhesives.

Fish gelatin is a biopolymer obtained by the hydrolysis of fish collagen [186]. It is rich in amino acids and can be used as a nutritional supplement. The natural characteristics of fish gelatin render its products relatively harmless to the body compared to clinical therapies and drugs [187]. Therefore, eating fish gelatin may have excellent benefits in patients with chronic diseases, such as hypertension, osteoporosis, and diabetes. Similar to fish collagen, fish gelatin is a potential alternative to mammalian gelatin. In addition, researchers have found a variety of drug delivery methods for fish gelatin products, such as external use or injection [188].

Gelatin extracted from fish by-products is mainly hydrolyzed using the acid method. It is generally believed that the yield and quality of gelatin obtained by hydrolysis are optimal at pH 4 [34]. Recently, gelatin extraction from swim bladders has been attempted using alkalis, enzymes, and high temperatures. For example, Kaewdang et al. used alkaline pretreatment with different Na_2_CO_3_:NaOH ratios (9:1, 8:2, 7:3, and 6:4) to extract fish glue from the swim bladder of *Thunnus albacores* and compared the yields obtained at these ratios [33]. The alkaline mixture (Na_2_CO_3_:NaOH) had a concentration of 4% (w/v). The results showed that the main components of all gelatins were α-chains. The Fourier transform infrared spectrum of gelatin showed that the molecular order of its triple-helical structure was lost. When the Na_2_CO_3_:NaOH ratio was 7:3, the yield of fish gelatin was the highest, up to 35.96%, with the highest imino acid content and gel strength. Compared with the gelatin obtained under other conditions, that obtained under the Na_2_CO_3_:NaOH ratio of 7:3 had a finer microstructure and smaller pores [33].

Zarubin et al. reported a microscopic process of extracting fish gelatin from by-products such as swim bladders by enzyme treatment [32]. First, the by-product samples of frozen fish (Gadidae) were initially treated three times with 10% citric acid aqueous solution and stirred continuously for 60 min. After washing, the samples were frozen at 25 ℃ for 2 h and then crushed. Then, the samples were heated in water (volume ratio of sample to water: 1:2) at 80–90 °C for 20–25 min and cooled to 35–40 °C. Next, a mixture of enzyme agents, "food collagenase" (produced by crab hepatopancreas, which is used for collagen hydrolysis) and "protease" (an animal-derived protease complex used in raw meat processing), was used to hydrolyze the material at 40 °C for 4 h, in which the weight ratio of enzyme mixture to sample was 1:1000. The mixture of the two agents could hydrolyze proteins in connective tissue and muscle tissue. The enzyme mixture was inactivated by heating at 70 °C for 15 min, and the broth was decanted. Finally, an ultra-permeable membrane made of aromatic polysulfonamide was used for ultrafiltration and the fish collagen was dried at 50–60 °C to obtain a dry powder. The approximate yield was 18 ± 2% [32].

Kaewdang et al. attempted to extract fish collagen using high-temperature treatment [34]. They used swim bladder from *Thunnus albacores* as a raw material to extract fish gelatin at 60, 70, and 80 °C, which resulted in extraction rates of 35.6%, 41.1%, and 47.3%, respectively, on a dry weight basis. The study showed that the amino acid composition of each gelatin was similar, mainly comprising glycine, and the imino acid residue was 169–172/1000. The molecular weight of gelatin decreased with increasing extraction temperature. Therefore, the gel strength of gelatin extracted at low temperatures was higher than that of gelatin extracted at high temperatures [34]. The gelling temperature of fish bladder gelatin ranged from 11.07 to 15.24 °C, while the melting temperature ranged from 20.36 to 22.33 °C. The authors also found that gelatin extracted at a lower temperature had a higher melting point. The microstructure of gelatin extracted at 60 °C was finer than that extracted at other temperatures [34].

##### Representative application: hydrogel

Soft supporting tissues such as cartilage and ligaments are the connective tissues in the body. Once damaged, these soft tissues cannot regenerate spontaneously in vivo [187, 190]. Hydrogels are soft and wet materials that have many similarities to biological tissues and can be a promising next-generation biological material for the treatment of soft tissue injury [191]. Collagen is the main rigid component of soft connective tissue and is arranged in various layers. SBC extracted from the sturgeon swim bladder has excellent characteristics, such as high denaturation temperature (Td), high solubility, uniformity, and low viscosity in acidic solutions [181, 182]. The Td of SBC in acidic and sodium phosphate buffer (pH = 7.2) solutions is 32.9 °C [180] and 43.0 °C [181], respectively, which is better than those of collagen sourced from some other aquatic species, such as salmon (18.6 °C) [192], grasscarp (24.6 °C) [193], starfish (24.7 °C) [194], jellyfish (28.9 °C) [195], shark (30.0 °C) [196], and tilapia (32.0 °C) [197]. The Td of SBC is equivalent to that of traditional animal collagen, including those from calf (37.0 °C) [181], rat tail (38.5 °C) [198], and porcine skin (41.3 °C) [181]. SBC can also form large fiber bundles at a very fast rate under certain conditions [181, 182]. Furthermore, SBC formed a gel with good mechanical stability. Because it is sourced from fish, the characteristics of SBC and lack of zoonosis provide great advantages for the manufacture of SBC-derived biomaterials [192–197]. These special properties of SBC make it an appealing candidate for developing hydrogels with anisotropic superstructures.

**Fig. 12:**
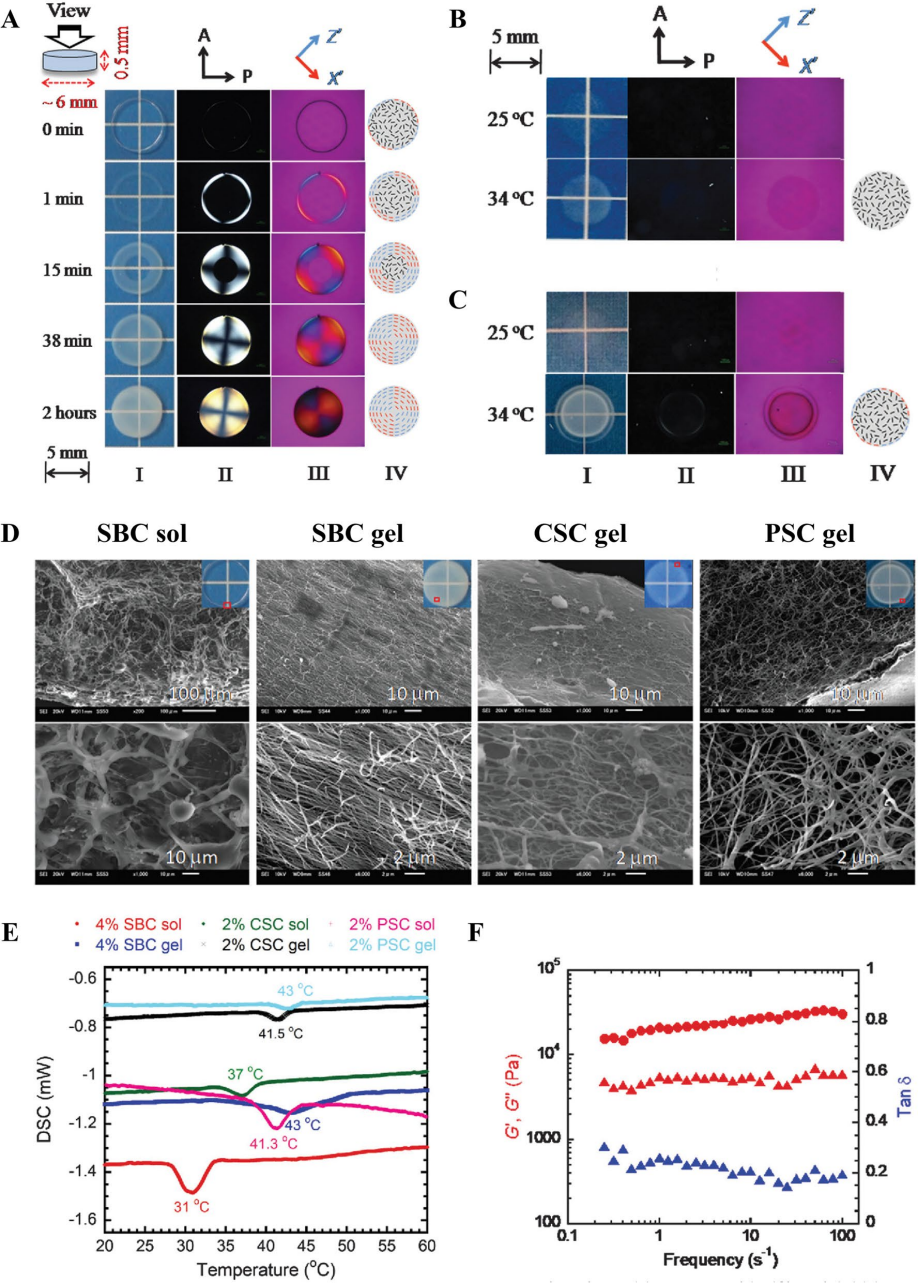
Performance comparison of SBC hydrogel (SBC gel) with calf skin collagen hydrogel (CSC gel) and porcine skin collagen hydrogel (PSC gel). Photographic and polarizing microscope images (POM) of SBC gel (**A**); photographic and POM images of CSC gel (**B**); photographic and POM images of PSC gel (**C**); SEM images of SBC solution (SBC sol), SBC gel, CSC gel, and PSC gel (**D**); differential scanning calorimetry (DSC) results of SBC sol, SBC gel, CSC solution (CSC sol), CSC gel, PSC solution (PSC sol), and PSC gel (**E**); rheological test results of SBC gel (**F**). Reprinted with permission from Ref. [181]. Copyright 2015, Royal Society of Chemistry

Mredha et al. found that type I collagen extracted from the swim bladder of the Bester sturgeon has excellent characteristics of high denaturation temperature, high solubility, and low viscosity, and can form large bundles of fibers very quickly under certain conditions [181]. With these characteristics, stable disc-shaped hydrogels with a concentric orientation of collagen fibers could be prepared at room temperature through the controlled diffusion of neutral buffer in the collagen solution, as shown in Fig. 12A. Traditional animal-derived collagen, such as calf skin collagen (CSC) and porcine skin collagen (PSC), could not form stable directional structures using this method (Fig. 12B, C). The authors believed that the rapid fibrillation rate of SBC led to rapid extrusion of the solvent from the gel phase to the sol phase during gelling, resulting in internal stress at the gel-sol interface [181]. Furthermore, this stress induced collagen molecules in the gel phase to arrange along the gel-sol interface, thus forming a concentric ring orientation. However, animal collagen solution was not conducive to the formation of this ordered structure because of its high viscosity and slow fiber-forming speed. SEM images revealed that the fibers of the SBC hydrogel (SBC gel) had a typical orientation arrangement, while those of the CSC hydrogel (CSC gel) and PSC hydrogel (PSC gel) lacked orientation arrangement (Fig. 12D). Compared with those of CSC and PSC before and after gel formation, the denaturation temperature (Fig. 12E) and storage modulus (Fig. 12F) of the SBC gel were significantly better [181]. Therefore, compared with animal-derived collagen hydrogel materials, SBC gels have better thermal and mechanical stability and are expected to be used in tissue repair such as cartilage and ligaments.

On this basis, Mredha et al. combined SBC with biocompatible poly (N, N'- dimethyl acrylamide) (PDMAAm) and successfully developed a new ductile hydrogel with anisotropic collagen fiber, based on the concept of a double network [29]. This double-network hydrogel (SBC/PDMAAm) was composed of physically and chemically crosslinked anisotropic SBC fibers as the first network and neutral PDMAAm as the second network. Using the excellent fiber-forming ability of SBC, the anisotropic structure of the SBC fiber network with good retention in the double-network hydrogel was formed by free injection. The extremely fast fibril-forming ability of SBC leads to hydrogel formation immediately after injection. The main structure was formed by injecting an acidic SBC solution into a Na_2_HPO_4_ solution with a pipette. Two types of fiber orientation patterns were formed in the SBC hydrogel. When collagen was rapidly injected into the salt solution (shear rate ~ 75 s^−1^), collagen molecules arranged owing to the injection shear form arranged fibrils. Then, because of the radial diffusion of the salt solution from the surface of the cylinder to the internal area and dehydration induced by fiber formation, some collagen molecules distorted and formed concentrically oriented fibrils. The anisotropic SBC hydrogel was immersed in a DMAAm solution, and polymerization of DMAAm was carried out. Finally, a collagen-based anisotropic double-network hydrogel (SBC/PDMAAm) with high toughness was obtained. The existence of an anisotropic fiber network structure was confirmed by SEM analysis of the circumferential and axial sections of the SBC/PDMAAm hydrogel. Furthermore, the DSC showed that the double-network hydrogel increased the denaturation temperature of collagen. The SBC/PDMAAm hydrogel showed good biomechanical properties in vivo after four weeks of implantation into the rabbit knee with osteochondral defects. Additionally, the hydroxyapatite-coated double-network hydrogel was tightly bound to the bone after four weeks [29]. This confirms that the fabricated collagen-based composite double-network hydrogel, as a soft and elastic ceramic material, not only shows excellent mechanical properties equivalent to those of natural cartilage but also has strong bone-bonding ability, which is expected to provide more choices for the design of next-generation orthopedic implants, such as artificial cartilage and bone defect repair materials in human weight-bearing areas.

##### Representative application: biological adhesive or glue

High-performance adhesives have been widely used in many high-technology fields [199–202]. Advances have been made in biological adhesives [203, 204] and high-performance synthetic adhesives including cyanoacrylate [205–207], polysaccharides [208–210], epoxy resins [211], polyurethane [212], polyvinyl acetate [213], phenolic [214], and adhesive hydrogels [215, 216]. However, these adhesives do not meet the requirements of medical adhesives effectively, and there are several limitations to their application in biomedicine. For example, cyanoacrylate has the disadvantages of weak tissue adhesion, poor degradation, and potential toxicity. In addition, it may cause an acute inflammatory reaction or tissue necrosis because of the heat generated during the curing process [207, 217]. In contrast, although fibrin-based adhesives (a type of biological adhesives) exhibit good biocompatibility, their bonding strength is relatively low [218]. Although mussel-based biological adhesives exhibit superior bonding performance [219], their cumbersome synthesis and biocompatibility limit their clinical application [220]. To overcome these drawbacks, the use of ready-made raw materials from natural resources to prepare biocompatible and biodegradable adhesives with strong adhesion properties is an attractive solution [221]. As early as several centuries ago, people found that fish glue extracted from swim bladders could be used to stick wood. Therefore, the use of fish glue may be an alternative strategy for developing powerful biological adhesives for biomedical applications. Swim bladders are collagen-based biomaterials that have excellent biocompatibility and biodegradability, and their highly organized 3D network structure results in high mechanical strength [185]. Therefore, the development of swim bladder-derived biological adhesives or glues has important biomedical applications and are expected to be a good candidate material for wound healing and tissue engineering.

**Fig. 13:**
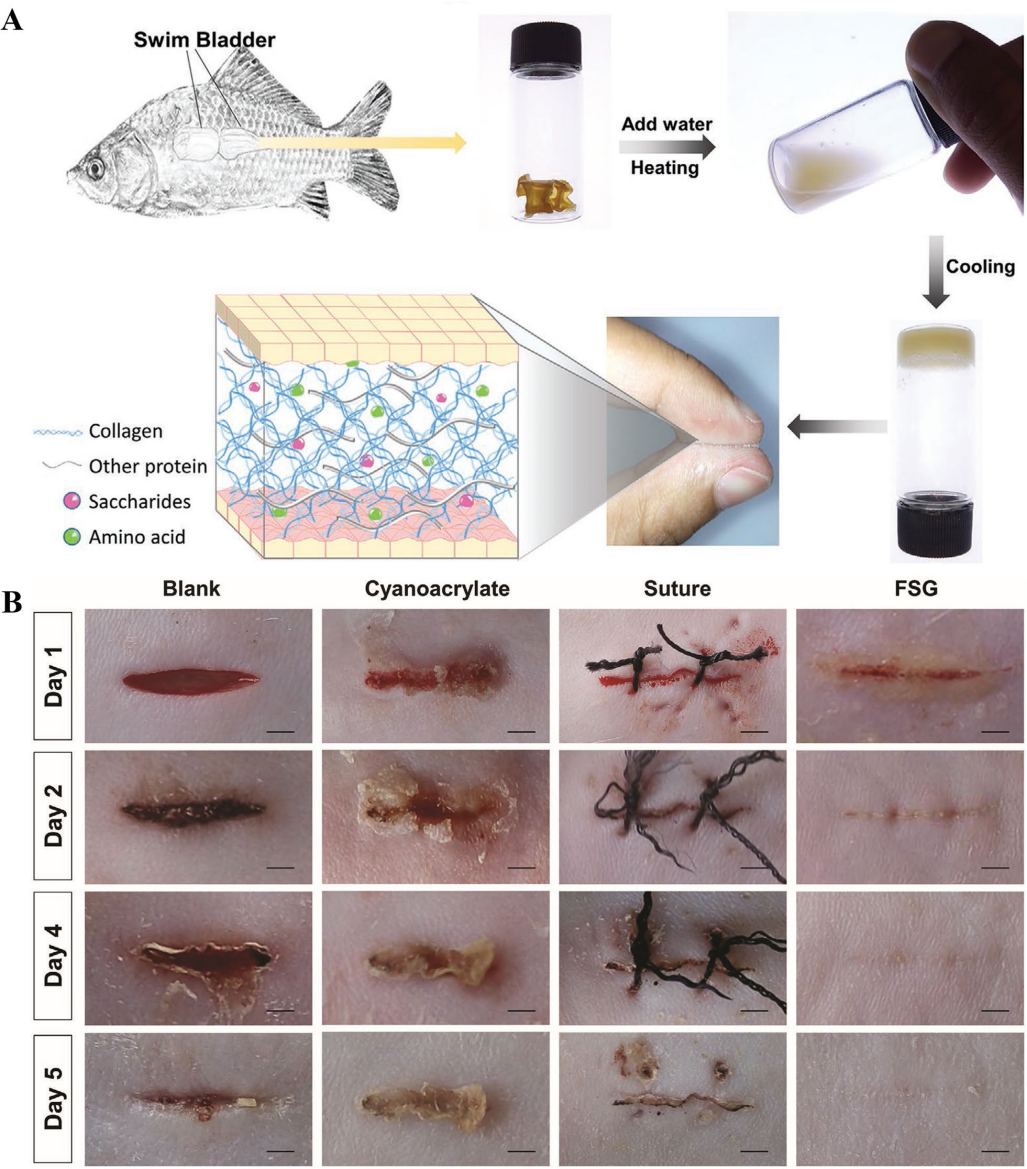
Preparation and bonding effect of fish swim bladder glue (FSG): Schematic diagram of preparation and mechanism (**A**), adhesive property of FSG on rat skin, and the effect of promoting wound healing (**B**). Reprinted with permission from Ref. [36]. Copyright 2021, Wiley–VCH GmbH

Xiao et al. successfully prepared a tough protein-based adhesive from dried swim bladder as raw material by simple heating and cooling treatment, followed by cutting the material into small pieces, and grinding into a powder with a mortar and pestle (Fig. 13A) [36]. The experimental results showed that the fish swim bladder glue (FSG) had excellent lap shear strength on various hard substrates (especially wood and glass) because of its highly crosslinked network structures and the strong interactions between collagen molecules of the FSG and substrate surface (Fig. 13A), which exceeded those of many commercial adhesives and artificial protein-based glues. In addition, FSG had low cytotoxicity and a small inflammatory response, and showed excellent adhesion performance in soft tissue. The biocompatibility and biodegradability of FSG, as well as its strong adhesion performance, suggest its ability to accelerate wound healing and skin regeneration (Fig. 13B). However, the main problem with fish glue is that it is easily polluted by microorganisms, such as bacteria, which is not conducive to its long-term storage. In this regard, Pan et al. used heat treatment and enzymatic hydrolysis to prepare fish bladder-derived glue, and then added antibacterial agents, such as borax, sodium diacetate, and anti-AL-D (an organic/inorganic composite antibacterial agent) [37]. The results showed that the three antibacterial agents effectively inhibited the growth of *Alternaria alternata* in the two fish bladder-derived glue samples. However, the use of different antibacterial agents had different effects on adhesion. It was preliminarily confirmed that the addition of anti-AL-D had the least effect on the shear strength of bonded wood components [37]. Therefore, the development of fish bladder-derived glue with long-lasting antibacterial effects can greatly improve biosafety in the storage process, which will help to further expand its use in the biomedical field.

However, some limitations remain in the development of swim bladder collagen or gelatin. For example, the differences in collagen content and composition in different species of swim bladders result in the different mechanical properties and thermal stability of the extracted collagen or gelatin materials. In addition, collagen or gelatin extraction methods commonly used for swim bladders use acids, bases, or organic solvents, which can cause environmental pollution, limiting the practical applications of collagen or gelatin extracted from swim bladder.

## Conclusion and outlook

Swim bladder-derived biomaterials are rich in collagen, elastin, and polysaccharides. Therefore, they have been widely used in various biomedical applications. When used directly as an ECM for tissue repair, dural repair, cardiovascular repair, and edible and pharmaceutical fish maw, the mechanical and biological properties of swim bladder, such as biocompatibility and anti-immunogenicity, are similar to or superior to those of tissue membranes of mammalian origin. In addition, the viscoelastic properties are very important features of biomaterials that interact with cells in regenerative medicine applications [222]. Like most soft tissue-derived materials, SBC is viscoelastic [89, 223–225] and can provide a suitable environment for cell growth and proliferation [224, 225]. Existing studies have mainly focused on the elastic modulus, strength, and other mechanical properties of swim bladder-derived materials [85–87, 89–91], while those on the interaction between viscoelasticity of swim bladder-derived biomaterials and cells are scarce; hence, this should be a focus area in future studies. When used indirectly, extracted collagen peptides, collagen or gelatin can be used for edible and pharmaceutical substances, or to prepare hydrogels or biological adhesives/glue with excellent comprehensive properties; such materials have abundant and broad biomedical applications. In retrospect, the research and application of swim bladder-derived biomaterials have just begun. In the future, the fabrication and modification of these biomaterials should be extensively improved. An increasing number of researchers are involved in this field, driven by the trend that biomedical materials have changed from traditional simple, physical functional substitutions to tissue regeneration and reconstruction. The future application of such materials in soft tissue, dural, and cardiovascular repair, along with other fields, will fully mobilize the healing process through various modes of regeneration and reconstruction of damaged human tissues or organs; these materials will also be able to restore and enhance the biological function and realize the permanent rehabilitation of damaged tissues or organs. Coupled with the naturally advantageous properties of swim bladder and its constituents, the development and use of swim bladder-derived biomaterials in biomedical therapeutics will become more prominent.

## Data Availability

All data are available in the paper.
